# Interneuronal DISC1 regulates NRG1-ErbB4 signalling and excitatory–inhibitory synapse formation in the mature cortex

**DOI:** 10.1038/ncomms10118

**Published:** 2015-12-11

**Authors:** Saurav Seshadri, Travis Faust, Koko Ishizuka, Kristen Delevich, Youjin Chung, Sun-Hong Kim, Martis Cowles, Minae Niwa, Hanna Jaaro-Peled, Toshifumi Tomoda, Cary Lai, E. S. Anton, Bo Li, Akira Sawa

**Affiliations:** 1 Department of Psychiatry, Johns Hopkins University, 600 North Wolfe Street, Meyer 3-166A, Baltimore, Maryland 21287, USA; 2 Department of Neuroscience, Johns Hopkins University, Baltimore, Maryland 21287, USA; 3 Watson School of Biological Sciences, Cold Spring Harbor Laboratory, Cold Spring Harbor, New York 11724, USA; 4 Department of Cell Biology and Physiology, University of North Carolina, Chapel Hill, North Carolina 27599, USA; 5 Department of Neurosciences, Beckman Research Institute of City of Hope, Duarte, California 91010, USA; 6 Department of Psychological and Brain Sciences, Indiana University, Bloomington, Indiana 47405, USA

## Abstract

Neuregulin-1 (NRG1) and its receptor ErbB4 influence several processes of neurodevelopment, but the mechanisms regulating this signalling in the mature brain are not well known. DISC1 is a multifunctional scaffold protein that mediates many cellular processes. Here we present a functional relationship between DISC1 and NRG1-ErbB4 signalling in mature cortical interneurons. By cell type-specific gene modulation *in vitro* and *in vivo* including in a mutant *DISC1* mouse model, we demonstrate that DISC1 inhibits NRG1-induced ErbB4 activation and signalling. This effect is likely mediated by competitive inhibition of binding of ErbB4 to PSD95. Finally, we show that interneuronal DISC1 affects NRG1-ErbB4-mediated phenotypes in the fast spiking interneuron-pyramidal neuron circuit. Post-mortem brain analyses and some genetic studies have reported interneuronal deficits and involvement of the *DISC1*, *NRG1* and *ErbB4* genes in schizophrenia, respectively. Our results suggest a mechanism by which cross-talk between DISC1 and NRG1-ErbB4 signalling may contribute to these deficits.

## Abstract (web summary)

Neuregulin-1 and DISC1 signalling pathways have both been linked to neurodevelopment and schizophrenia. Here, Seshadri *et al.* demonstrate that DISC1 negatively regulates NRG1-induced ErbB4 signalling in adult cortical interneurons both *in vitro* and *in vivo*, possibly via competitive binding to PSD95.

## Graphical abstract

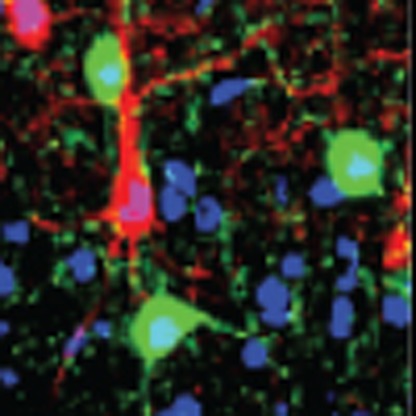

Classic linkage/association and copy number variations studies have suggested that neuregulin-1 (NRG1) and its receptor ErbB4 are implicated in psychiatric disorders^1^,^2^,^3^,^4^. NRG1-ErbB4 signalling was initially studied in the context of neurodevelopment. This cascade is important in guiding several aspects of cortical maturation including radial migration of pyramidal neurons, tangential migration of interneurons and myelination^5^.

In the cerebral cortex of the postnatal brain, NRG1-ErbB4 signalling enhances excitatory synapse formation on interneurons and inhibitory synapse formation on pyramidal neurons^6^,^7^,^8^. In the cortex, ErbB4 is primarily expressed in interneurons, particularly in parvalbumin (PV)-positive interneurons^6^,^9^,^10^. At the electrophysiological level, NRG1-ErbB4 signalling increases miniature excitatory postsynaptic current (mEPSC) frequency and amplitude in interneurons, and increases miniature inhibitory postsynaptic current frequency in pyramidal neurons^6^,^11^,^12^. Interneuronal ErbB4 also affects dendritic spine density and excitatory synapse formation in pyramidal neurons^7^,^13^. These results show that NRG1 signalling, via ErbB4 in interneurons, plays a critical role in regulating excitatory–inhibitory (*E*–*I*) balance in the cortex. Given that the *E*–*I* imbalance may underlie the pathophysiology of mental disorders such as schizophrenia^14^,^15^, it is crucial to elucidate the mechanism(s) by which NRG1-ErbB4 signalling is regulated in interneurons of the adult cortex.

The gene coding for DISC1 was originally identified at the breakpoint of a balanced translocation co-segregated with mental illnesses including schizophrenia and major depression in a large Scottish pedigree^16^. Biological studies exploring the physiological roles of DISC1 have demonstrated that it is crucial in neurodevelopment and neural signalling in the adult brain^17^,^18^,^19^. Although most of these studies have focused on pyramidal neurons in the cortex and newborn neurons in the dentate gyrus^20^,^21^,^22^,^23^, DISC1 is also expressed in cortical interneurons^24^,^25^. Accordingly, recent studies in mice carrying a point mutation in the *Disc1* gene or knockdown of *Disc1* by short hairpin RNA (shRNA) have shown deficits in interneuronal migration and positioning during development^26^,^27^. However, the function of DISC1 in interneurons in the mature cortex remains to be elucidated.

In the present study, we provide evidence that DISC1 modulates NRG1-ErbB4 signalling in interneurons. The protein interaction of ErbB4 and postsynaptic density protein-95 (PSD95) is critical to NRG1-ErbB4 signalling^28^,^29^. In addition, DISC1 is known to interact with and regulate PSD95-associated proteins^20^. Here we suggest that the influence of DISC1 on NRG1-induced ErbB4 activation is likely mediated by competitive inhibition of binding of ErbB4 to PSD95. We directly demonstrate that selective modulation of interneuronal DISC1 affects NRG1-ErbB4-mediated phenotypes at the fast spiking interneuron-pyramidal neuron circuit level, which in turn regulates *E*–*I* circuitry in the mature cortex.

## Results

### Interneuronal DISC1 regulates ErbB4 signalling *in vitro*

Both DISC1 and ErbB4 interact with PSD95 (refs 20, 28). ErbB4 is expressed in PV-positive interneurons in the cortex^6^,^10^. DISC1 is also reportedly expressed in interneurons^24^,^25^, and we confirmed its expression in PV-positive interneurons in the cortex (Supplementary Fig. 1a). We therefore hypothesized that DISC1 may influence NRG1-ErbB4 signalling via association with the PSD95 scaffold in interneurons. To test this, we first examined whether DISC1 knockdown affects NRG1-induced ErbB4 activation *in vitro*. As ErbB4 is a receptor tyrosine kinase, which undergoes phosphorylation upon activation by NRG1, phospho-ErbB4 (pErbB4) immunoreactivity can be used as an indicator of NRG1-ErbB4 signalling^30^. Green fluorescent protein (GFP) expression was similar across conditions (Supplementary Fig. 1b). Immunoreactivity to pErbB4 was observed in the cell body and processes (Supplementary Fig. 1c). The specificity of ErbB4 and pErbB4 antibodies was confirmed *in vitro* and *in vivo* (Supplementary Fig. 2a–d). Western blots detected by all major antibodies were quantified within their linear ranges (Supplementary Fig. 3a–c). Full-length versions of all blots are provided in Supplementary Figs 11 and 12. The epidermal growth factor-like domain of NRG1 is known to be necessary and sufficient for binding with ErbB4 and activation of NRG1-ErbB4 signalling^31^. Using primary cortical neuron cultures in which ∼20% cells are in interneuronal populations^32^, we confirmed that treatment with a recombinant protein consisting of the epidermal growth factor-like domain of NRG1 (hereafter referred to as NRG1) induced ErbB4 activation, detected by pErbB4 immunoreactivity (Supplementary Fig. 2d).

**Figure 1:**
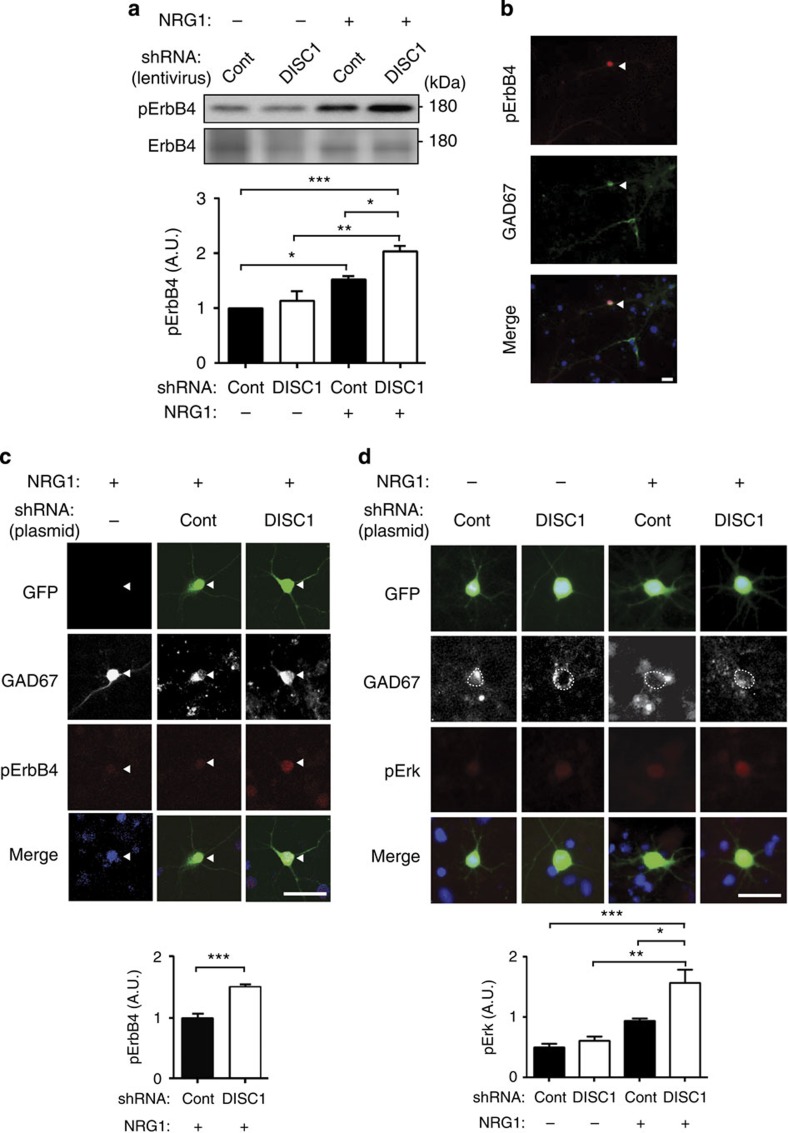
Interneuronal DISC1 knockdown enhances NRG1-induced ErbB4 activation and signalling in interneurons *in vitro.* (**a**) Western blot for ErbB4 and pErbB4 in lysates from mature primary cortical neurons infected with shRNA (Control (Cont) or DISC1) and treated with NRG1. Cells with DISC1 shRNA showed a greater NRG1-induced increase in pErbB4 signal. (**b**) Immunostaining for pErbB4 and GAD67 in mature primary cortical neurons. pErbB4 signal co-localized with GAD67 (arrowhead), confirming interneuron-specific expression of ErbB4. Ninety-one percent of pErbB4-positive (pErbB4^+^) cells were GAD67-positive (GAD67^+^; *N*=5 cultures). (**c**) Immunostaining for pErbB4 and GAD67 in mature primary cortical neurons, mock transfected or transfected with shRNA (Cont or DISC1, GFP co-expressed) and treated with NRG1. GAD67-positive interneurons with DISC1 knockdown showed greater NRG1-induced pErbB4 immunoreactivity (arrowheads). (**d**) Immunostaining for pErk and GAD67 in mature primary cortical neurons transfected with shRNA (Cont or DISC1, GFP co-expressed) and treated with NRG1. GAD67-positive interneurons with DISC1 shRNA showed a greater NRG1-induced increase in pErk immunoreactivity. Blue signal in images represents 4,6-diamidino-2-phenylindole stain. *N*>25 cells per condition. Data are represented as mean±s.e.m. *P<0.05, **P<0.01, ***P<0.001; scale bars, 10 μm.

To test the role of DISC1 on NRG1-ErbB4 signalling, we then used lentiviral infection to express control or DISC1 shRNA in primary neuron cultures, followed by NRG1 treatment. This approach results in DISC1 knockdown in a majority of cells (Supplementary Fig. 4a), a necessary proportion for observing changes at the biochemical level. We found that the level of pErbB4 was significantly augmented by NRG1 treatment (5 nM, 15 min), whereas total ErbB4 expression was unchanged (Fig. 1a). This augmentation was significantly greater in cells expressing DISC1 shRNA compared with those with control shRNA, suggesting that DISC1 knockdown enhances activation of ErbB4 by NRG1 (Fig. 1a). We confirmed that this effect was reversed by lentiviral co-expression of shRNA-resistant DISC1 (‘DISC1 rescue'; Supplementary Fig. 4b). This experiment indicated that the enhancement of ErbB4 activation by DISC1 shRNA is not an off-target effect but is in fact mediated by knockdown of endogenous DISC1. These findings are consistent with previous studies demonstrating that DISC1 shRNA-induced phenotypes were specifically normalized by co-expression of shRNA-resistant DISC1 (refs 20, 33, 34, 35).

ErbB4 is expressed almost exclusively in interneurons^6^,^10^ and our mixed cultures contain a substantial interneuronal population^36^. We therefore interpret these biochemical data as an interneuron-specific enhancement of ErbB4 activation. To support this interpretation, we first confirmed that pErbB4 immunoreactivity was only detected in interneurons by observing co-localization of pErbB4 with glutamic acid decarboxylase (GAD67), an interneuronal marker. A majority of pErbB4-positive cells were GAD67 positive (91±1.8%, mean±s.e.m.; Fig. 1b). We then quantified the effect of DISC1 knockdown on NRG1-ErbB4 signalling by immunocytochemistry for pErbB4. We again found that NRG1-induced ErbB4 activation, measured by interneuron-specific pErbB4 immunoreactivity, was significantly greater in cells expressing DISC1 shRNA compared with those with control shRNA (Supplementary Fig. 4c). This increase was similar in magnitude to that observed in Fig. 1a, indicating that our biochemical results accurately reflect an interneuron-specific effect of DISC1 on ErbB4 activation.

To determine whether this enhancement of NRG1-ErbB4 signalling was a cell-autonomous effect of interneuronal DISC1, we expressed control or DISC1 shRNA and GFP in cultured primary neurons by plasmid transfection, rather than lentiviral infection as above. By virtue of the very low transfection efficiency of the reagent used (less than 5–10%), shRNA-expressing neurons in transfected cultures are surrounded by non-transfected cells in which DISC1 is unaffected (Supplementary Fig. 5a). Thus, functional interactions between cells with DISC1 knockdown are negligible and changes observed in DISC1 shRNA-expressing neurons likely reflect cell-autonomous effects of DISC1. Cultures were treated with NRG1 and co-stained for pErbB4 and GAD67, in order to restrict analysis to transfected interneurons. In this experimental condition, ErbB4 activation (indicated by pErbB4 immunoreactivity) following NRG1 treatment was significantly higher in cells expressing DISC1 shRNA compared with control shRNA (Fig. 1c). This indicates that increased ErbB4 activation is a result of reduced interneuronal DISC1. Finally, we used the same experimental system to assess whether NRG1-induced phosphorylation of extracellular signal-regulated kinase (Erk), a downstream effector of ErbB4 (ref. 5), was also enhanced by DISC1 knockdown. Erk phosphorylation was significantly increased in GAD67-positive cells expressing DISC1 shRNA compared with control shRNA (Fig. 1d). Erk phosphorylation was not significantly changed in GAD67-negative cells, indicating that this effect on NRG1-ErbB4 downstream signalling is a cell autonomous and interneuron-specific effect of DISC1 (Supplementary Fig. 5b).

### Interneuronal DISC1 regulates ErbB4 signalling *in vivo*

**Figure 2:**
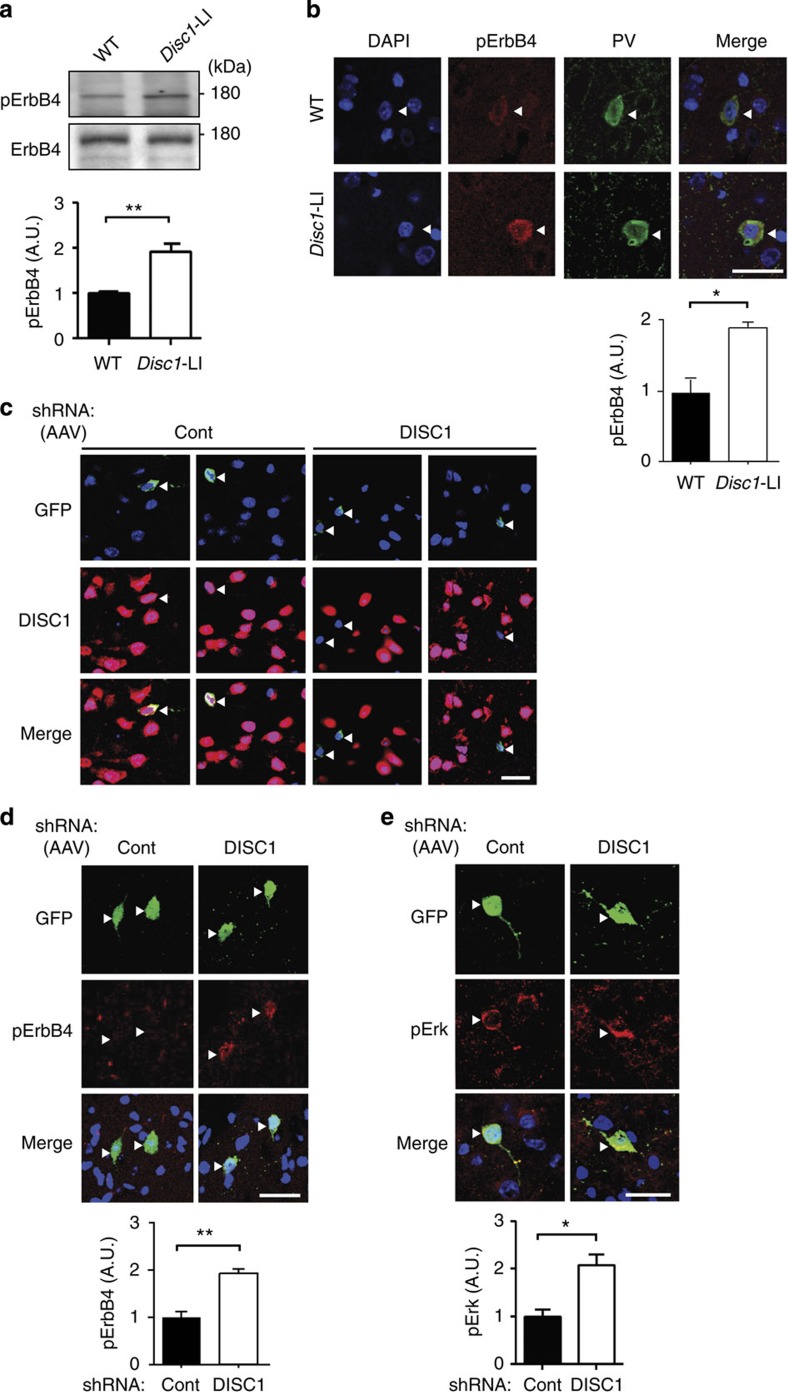
Interneuronal DISC1 knockdown enhances ErbB4 activation and signalling in interneurons *in vivo.* (**a**) Western blot for ErbB4 and pErbB4 in cortical lysates from adult *Disc1*-LI mice or WT littermates. *Disc1*-LI mice showed greater pErbB4 signal. (**b**) Immunostaining for pErbB4 in cortical sections (layer 3, PFC) from adult *Disc1*-LI mice or WT littermates. *Disc1*-LI mice showed greater pErbB4 immunoreactivity in PV-positive cells (arrowheads). Scale bar, 10 μm. (**c**) Immunostaining for DISC1 in cortical sections (layer 3, PFC) from PV-Cre mice injected with AAV expressing Cre-dependent shRNA (Control (Cont) or DISC1) and GFP. Images from two independent and representative fields are shown. DISC1 expression is knocked down exclusively by DISC1 shRNA, specifically in GFP-positive cells (arrowheads). Scale bar, 10 μm. (**d**) Immunostaining for pErbB4 in PV-Cre mice injected with AAV expressing Cre-dependent shRNA (Cont or DISC1) and GFP. pErbB4 is increased in GFP-positive cells expressing DISC1 shRNA (arrowheads). Scale bar, 10 μm. (**e**) Immunostaining for pErk in PV-Cre mice injected with AAV-expressing Cre-dependent shRNA (Cont or DISC1) and GFP. pErk is increased in GFP-positive cells expressing DISC1 shRNA (arrowheads). Blue signal in images represents 4,6-diamidino-2-phenylindole (DAPI) stain. *N*>30 cells from five mice. Data are represented as mean±s.e.m. *P<0.05, **P<0.01; scale bar, 10 μm.

To address whether knockdown of DISC1 also enhances ErbB4 activation *in vivo*, we first used a *Disc1* locus impairment (LI) model, in which the majority of *Disc1* isoforms are abolished (Supplementary Fig. 6)^37^. A significant increase in activation of ErbB4 was observed at the biochemical level in cortical extracts from *Disc1-*LI mice compared with those from wild-type (WT) littermates (Fig. 2a). This observation was validated by immunohistochemistry for pErbB4, an established marker for ErbB4 activation (Fig. 2b). *Disc1*-LI mice did not show a significant difference in total numbers of PV interneurons in the cortex (Supplementary Fig. 7). Expression of NRG1 was unchanged in the *Disc1-*LI model, suggesting that this effect was more likely attributable to a postsynaptic change in regulation of existing NRG1-ErbB4 signalling, rather than an overall increase in NRG1 signalling (Supplementary Fig. 8a). As pErbB4 immunoreactivity is restricted to PV interneurons *in vivo* (Supplementary Fig. 8b), this effect is likely attributable to PV interneurons.

To address whether this effect was cell-autonomous, we knocked down DISC1 in a PV-positive interneuron-specific manner. This was accomplished using an adeno-associated virus (AAV) that expresses control or DISC1 shRNA, together with GFP, downstream of a floxed stop codon. AAV was stereotaxically injected into the cortex of PV-Cre mice, resulting in selective expression of shRNA and GFP in PV interneurons. The Cre-dependence and PV interneuron-specificity of GFP expression were verified *in vitro* and *in vivo,* respectively (Supplementary Fig. 8c–e). We confirmed that knockdown of DISC1 expression was restricted to GFP- and PV-positive interneurons in the mice (Fig. 2c). Consistent with *in vitro* results, we observed increased ErbB4 activation in interneurons following DISC1 knockdown (Fig. 2d). Downstream Erk signalling was also enhanced in these cells (Fig. 2e).

### DISC1 interferes with ErbB4–PSD95 interaction

**Figure 3:**
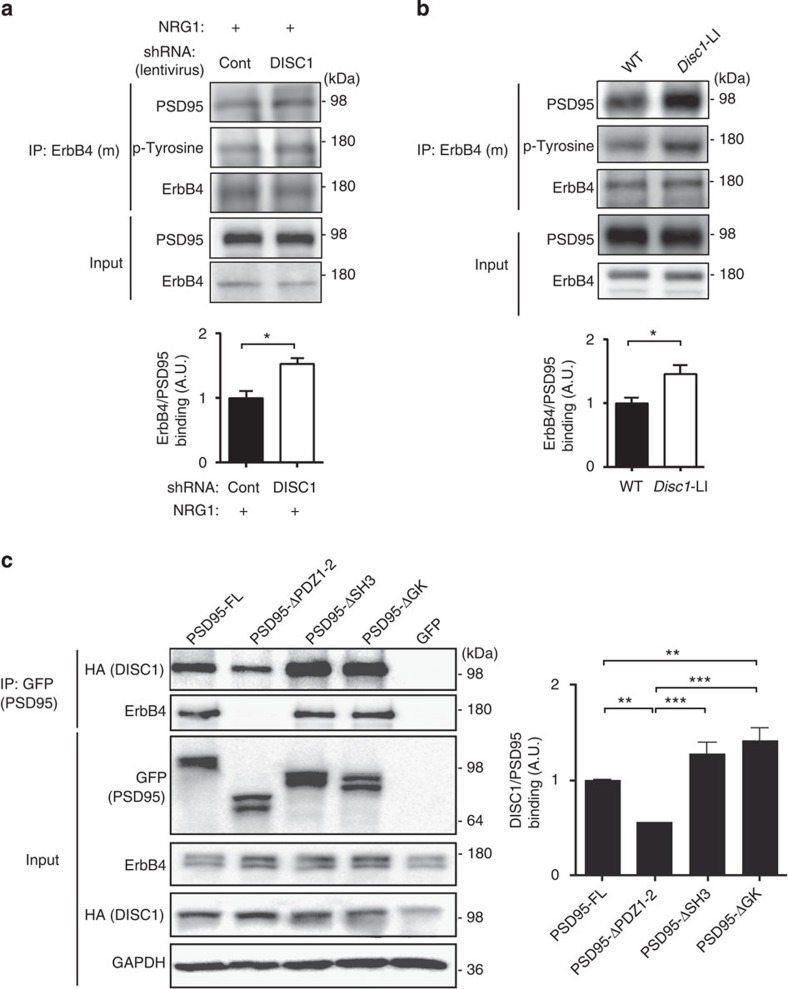
DISC1 interferes with ErbB4–PSD95 interaction. (**a**) Co-immunoprecipitation (co-IP) of ErbB4 and PSD95 in lysates from mature primary cortical neurons infected with shRNA (control (Cont) or DISC1) and treated with NRG1. Top panels, western blot for PSD95, p-tyrosine and ErbB4 in anti-ErbB4 precipitates; bottom panels, western blot for PSD95 and ErbB4 in input. Cells with DISC1 shRNA showed greater amounts of PSD95 co-precipitating with ErbB4 and greater levels of NRG1-induced tyrosine phosphorylation of ErbB4. (**b**) Co-IP of ErbB4 and PSD95 in cortical lysates from adult *Disc1*-LI mice or WT littermates. Top panels, western blot for PSD95, p-tyrosine and ErbB4 in anti-ErbB4 precipitates; bottom panels, western blot for PSD95 and ErbB4 in input. *Disc1*-LI mice showed greater amounts of PSD95 co-precipitating with ErbB4 and greater levels of NRG1-induced tyrosine phosphorylation of ErbB4. (**c**) Co-IP of PSD95, DISC1 and ErbB4 in lysates from HEK-293 cells. Cells were transfected with haemagglutinin (HA)-tagged DISC1, ErbB4 and GFP-tagged PSD95 constructs (full length (FL) or with the following deletions: PDZ domain 1 and 2 (ΔPDZ1-2), Src homology 3 domain (ΔSH3) and guanylate kinase-like domain (ΔGK)). Top panels, western blot for HA (DISC1) and ErbB4 in anti-GFP (PSD95) precipitates; bottom panels, western blot for PSD95, ErbB4, DISC1 and GAPDH in input. Amounts of DISC1 and ErbB4 co-precipitating with PSD95-ΔPDZ1-2 were significantly reduced compared with PSD95-FL. Data are represented as mean±s.e.m. *P<0.05, **P<0.01, ***P<0.01. *N*=3 independent experiments.

As described above, both ErbB4 and DISC1 have been reported to interact with PSD95 (refs 20, 28). ErbB4–PSD95 interaction facilitates NRG1-mediated activation of ErbB4 (ref. 29). We therefore hypothesized that DISC1 may affect ErbB4 activation by regulating its interaction with PSD95. To test this idea, we examined whether ErbB4-PSD95 binding is affected by DISC1 knockdown or genetic deletion of DISC1 isoforms. We found that ErbB4–PSD95 interaction was increased in primary neuron cultures infected with DISC1 shRNA virus compared with control shRNA-infected cells (Fig. 3a). This was shown by an increase of PSD95 in ErbB4 precipitates in which the immunoreactivity of p-tyrosine is also increased (indicating an increase of pErbB4, as described above; Fig. 3a). ErbB4–PSD95 interaction and ErbB4 activation were also augmented in cortical extracts from *Disc1-*LI mice compared with those from WT controls (Fig. 3b). These results suggest that DISC1 inhibits ErbB4-PSD95 binding *in vitro* and *in vivo*.

To assess whether DISC1 knockdown affects expression of PSD95 or trafficking to excitatory synapses, we measured synaptic PSD95 in cultured primary neurons transfected with control or DISC1 shRNA (Supplementary Fig. 9). As no significant changes in synaptic PSD95 levels were observed, we hypothesized that DISC1 may interfere with ErbB4 binding to PSD95 by a competitive mechanism. To address this possibility, we determined the binding domain of PSD95 for DISC1 using a series of PSD95 deletion constructs that were generated based on established domain structures of PSD95 (refs 38, 39; see Supplementary Methods for details). Deletion of the first two PDZ domains of PSD95, an area crucial for binding with ErbB4 (refs 28, 40), also significantly reduced PSD95 interaction with DISC1 (Fig. 3c). Thus, ErbB4 and DISC1 may compete with each other for PSD95 binding by the same domains.

**Figure 4:**
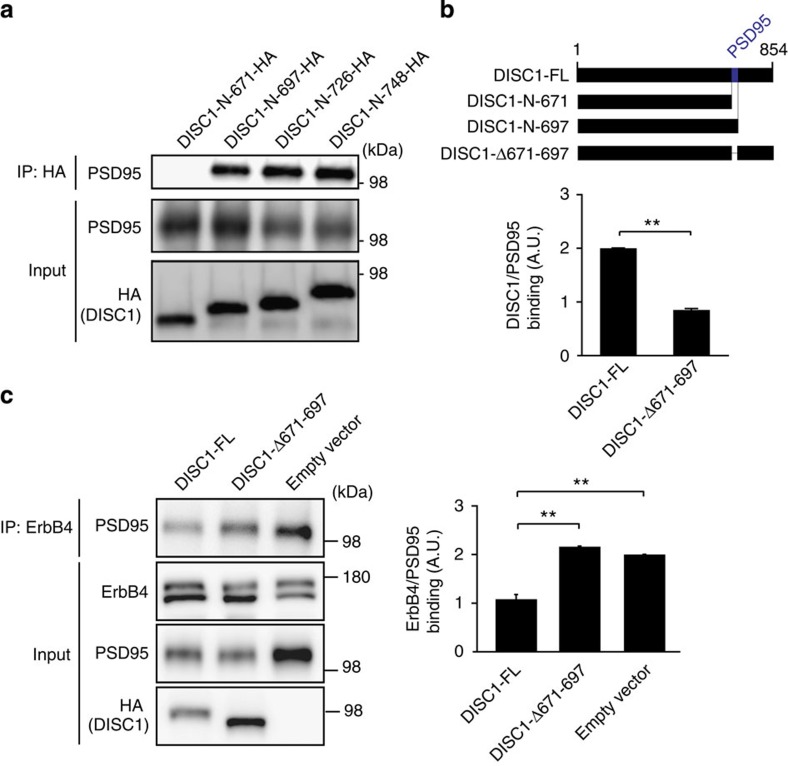
DISC1–PSD95 interaction may be important in regulating ErbB4–PSD95 interaction. (**a**) Co-IP of DISC1 and PSD95 in lysates from HEK-293 cells. Cells were transfected with GFP-tagged PSD95, ErbB4 and haemagglutinin (HA)-tagged truncated DISC1 constructs (N-terminal to amino acid 671 (N-671-HA), 697 (N-697-HA), 726 (N-726-HA) or 748 (N-748-HA)). Top panel, western blot for PSD95 in anti-HA (DISC1) precipitates; bottom panels, western blot for PSD95 and HA (DISC1) in input. Amounts of PSD95 co-precipitating with DISC1-N-671-HA were significantly reduced compared with DISC1-N-697-HA, suggesting AA 671–697 as the binding domain for PSD95. (**b**) Left panel, schematic of full-length DISC1 (FL), truncated DISC1 constructs and DISC1 deletion construct deficient in binding to PSD95 (DISC1-Δ671–697); right panel, quantification of co-IP of PSD95 and DISC1-Δ671–697 deletion construct. Binding of PSD95 to DISC1-Δ671–697 was significantly impaired. (**c**) Co-IP of ErbB4 and PSD95 in lysates from HEK-293 cells. Cells were transfected with ErbB4, PSD95 and HA-tagged DISC1 constructs (FL or lacking the binding domain for PSD95). Top panel, western blot for PSD95 in anti-ErbB4 precipitates; bottom panels, western blot for ErbB4, PSD95 and HA (DISC1) in input. Co-transfection of DISC1-FL impaired ErbB4-PSD95 binding, whereas DISC1-Δ671–697 did not. Data are represented as mean±s.e.m. **P<0.01. *N*=3 independent experiments.

To test the importance of DISC1–PSD95 interaction in the regulation of ErbB4–PSD95 interaction, we first determined the binding domain of DISC1 for PSD95 using a series of DISC1 deletion constructs. Protein product from a construct containing the first 697 amino acids from the N-terminus of DISC1 (DISC1-N-697) was able to bind to PSD95, whereas protein product from a construct containing the first 671 amino acids (DISC1-N-671) did not, implying that amino acids 671–697 of DISC1 are important in this interaction (Fig. 4a). Thus, we produced a construct lacking these amino acids (DISC1-Δ671–697) and found that deletion of this region significantly reduced DISC1-PSD95 binding (Fig. 4b). We found that while co-expression of full-length DISC1 significantly reduced ErbB4-PSD95 binding, co-expression of DISC1-Δ671–697 did not affect ErbB4-PSD95 binding (Fig. 4c). Taken together, our results suggest that DISC1 interferes with ErbB4-PSD95 binding via its own interaction with PSD95.

### Interneuronal DISC1 regulates *E*–*I* synapse formation

**Figure 5:**
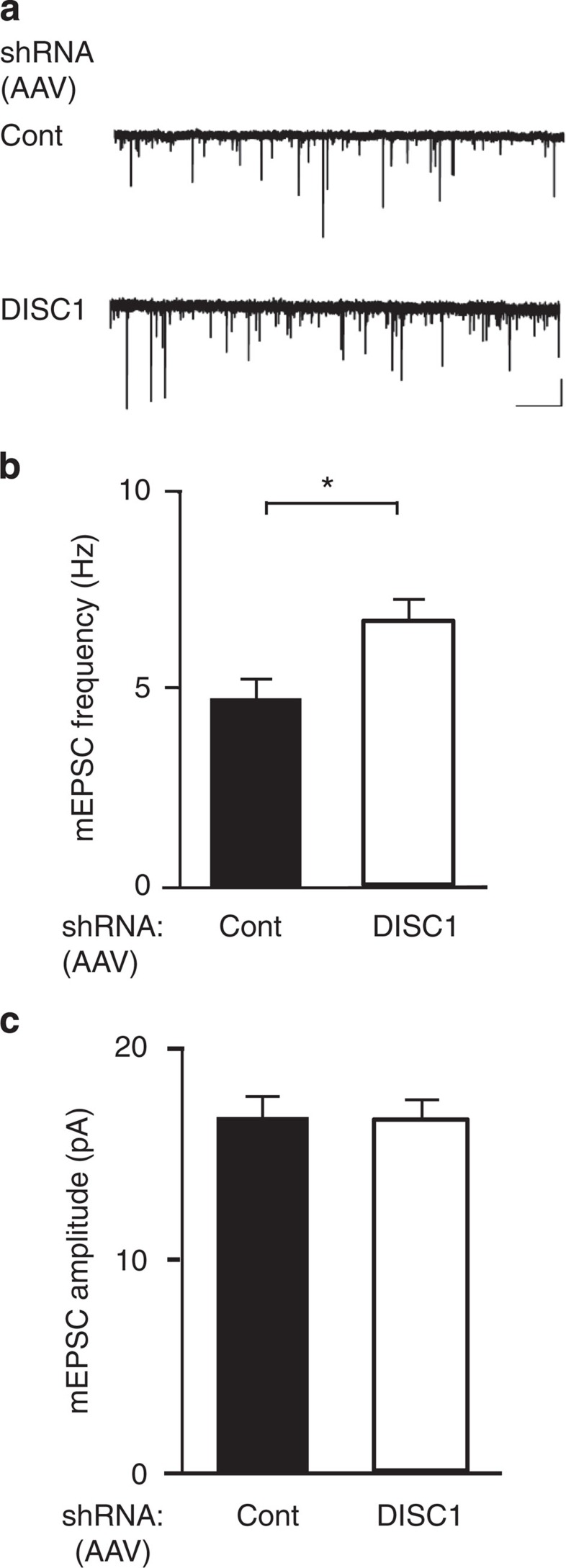
Interneuronal DISC1 knockdown increases interneuronal excitation. (**a**) Representative current traces from PV interneurons infected with shRNA (control (Cont) or DISC1) in acute cortical slices from adult mice recorded in the whole-cell configuration. Negative peaks represent mEPSCs. Scale bars, *x* axis, 500 ms, *y* axis, 20 pA. (**b**) Quantification of mEPSC frequency in recordings from PV interneurons infected with shRNA (Cont or DISC1). DISC1 knockdown led to an increase in mEPSC frequency. (**c**) Quantification of mEPSC amplitude in recordings from PV interneurons infected with shRNA (Cont or DISC1). DISC1 knockdown did not affect mEPSC amplitude. Data are represented as mean±s.e.m. *P<0.05. *N*=5 animals, 27 cells for DISC1 shRNA, 4 animals, 20 cells for Cont shRNA.

NRG1-ErbB4 signalling has been shown to enhance mEPSC frequency *in vitro*^6^,^11^,^12^. We therefore hypothesized that interneuronal knockdown of DISC1 may enhance mEPSC frequency, possibly as a downstream effect of enhanced NRG1-ErbB4 signalling. As above, we selectively knocked down DISC1 in fast-spiking interneurons by cortical injection of AAV with Cre-dependent shRNA expression into PV-Cre mice. GFP-positive cells (that is, infected PV interneurons) were patched in acute slices from these mice in order to record mEPSCs. We found that mEPSC frequency was increased in cells with DISC1 knockdown compared with cells expressing control shRNA (Fig. 5a, b), whereas amplitude of mEPSCs was not affected (Fig. 5c). DISC1 knockdown did not affect the membrane properties or intrinsic excitability of PV interneurons (Supplementary Fig. 10). These results indicate that DISC1 knockdown in interneurons enhances their excitability in response to pyramidal neurons through increased synapse formation and/or modification of pre-synaptic elements.

**Figure 6:**
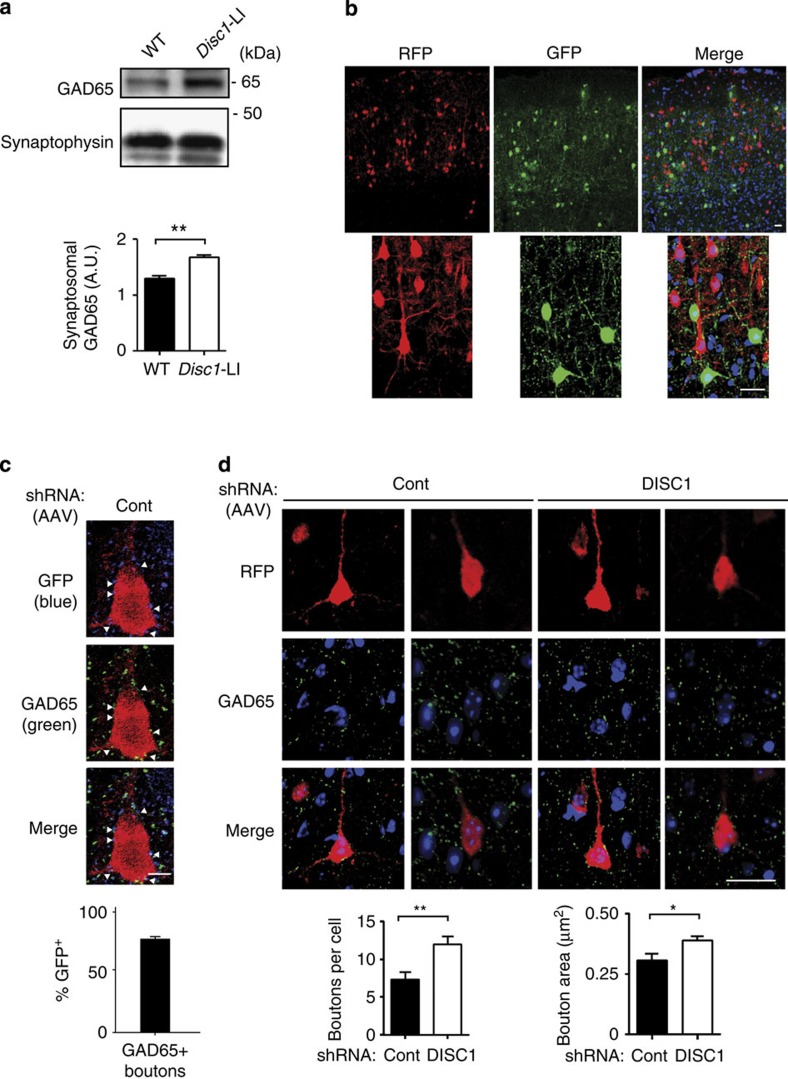
Interneuronal DISC1 knockdown increases inhibitory synapse formation on pyramidal neurons. (**a**) Western blot for GAD65 in synaptosomal fractions from adult *Disc1*-LI mice or WT littermates. *Disc1*-LI mice showed greater levels of synaptosomal GAD65. Synaptophysin was used as a loading control (Cont). (**b**) Immunohistochemistry showing lentiviral- and AAV-mediated cell type-specific fluorescent labelling. Pyramidal neurons and interneurons are individually labelled by RFP and GFP, respectively. (**c**) Immunohistochemistry for GFP (blue), GAD65 (green) and RFP (red) in PV-Cre mice injected with AAV. The majority (76%, *N*=13 cells) of GAD65-positive (GAD65^+^) boutons were also GFP-positive (GFP^+^), that is, originating from infected PV interneurons (arrowheads). Scale bar, 5 μm. (**d**) Top, immunohistochemistry for RFP (red, pyramidal neurons) and GAD65 (green) in PV-Cre mice with interneuronal shRNA expression (Cont or DISC1). GAD65^+^ punctae adjacent to RFP-labelled cell bodies, that is, perisomatic GAD65 boutons, were analysed. Bottom, quantification of GAD65 bouton number and size (area). DISC1 knockdown led to denser and larger perisomatic GAD65 boutons. Blue signal in images represents 4,6-diamidino-2-phenylindole (DAPI) stain (nucleus). *N*=14 cells from 4 mice. Data are represented as mean±s.e.m. *P<0.05, **P<0.01; scale bar, 20 μm.

NRG1-ErbB4 signalling in interneurons increases formation of glutamic acid decarboxylase (GAD65)-positive inhibitory boutons on the soma and axon initial segment of pyramidal neurons in the cortex^6^. This regulation of inhibitory connections is also reflected electrophysiologically by the effects of NRG1-ErbB4 signalling on miniature inhibitory postsynaptic current frequency in pyramidal neurons, in the cortex^6^,^41^,^42^ and amygdala^43^. We therefore examined whether loss of DISC1 expression, also enhanced this downstream effect of NRG1-ErbB4 signalling. Synaptosomal fractionation of cortical extracts showed that the level of synaptic GAD65 was increased in *Disc1*-LI mice compared with WT littermates (Fig. 6a). Previous studies showed no change in GAD65 expression following ErbB4 knockdown^7^,^8^. However, our results probably differ for two reasons. First, the present study functionally upregulated ErbB4, whereas the previous studies suppressed ErbB4. Second, the present study examined ErbB4 in synaptosomal fractions, whereas the previous studies used total extracts. To more accurately quantify GAD65-positive perisomatic inhibitory bouton formation on pyramidal neurons, we co-injected a lentivirus expressing red fluorescent protein (RFP) under the control of the pyramidal neuron-specific αCaMKII promoter along with the AAV used for PV interneuron-specific DISC1 knockdown described above (Fig. 6b). We then performed immunohistochemistry for GAD65 in cortical sections. The majority of GAD65-positive boutons were also GFP-positive, indicating that they come from infected PV interneurons (Fig. 6c). We observed a significant increase in both number and size of perisomatic GAD65-positive boutons following interneuronal DISC1 knockdown *in vivo* (Fig. 6d). As these boutons represent inhibitory output from PV interneurons to pyramidal neurons, these results suggest a novel circuit-level phenotype that is regulated by interneuronal DISC1.

## Discussion

The present work represents the first evidence of direct involvement of DISC1 in regulating NRG1-ErbB4 signalling in adult fast-spiking interneurons in a cell-autonomous manner. Our data suggest that DISC1 negatively regulates ErbB4 activation at excitatory synapses onto PV interneurons. We observed that DISC1 competed with ErbB4 for binding to PSD95, which might reduce both ErbB4-PSD95 binding and subsequent ErbB4 activation. This scenario is the most likely; however, we note that there is room for alternative interpretations. The regulation of ErbB4 signalling by DISC1 is likely to be an important contributor to maintaining excitatory–inhibitory balance in the healthy adult cortex. We observed that selective knockdown of interneuronal DISC1 affects two major characteristics of interneurons affected by NRG1-ErbB4 signalling, that is, interneuronal mEPSC frequency and inhibitory GAD65 bouton formation.

There are several technical limitations to this study. First, it is challenging to study each subpopulation of interneurons in primary neuron cultures, in part due to their low prevalence. For this reason, we used GAD67 rather than PV as an interneuronal marker for immunocytochemistry experiments, in order to maximize the number of transfected interneurons we could observe in each culture. We validated our ability to observe interneuronal ErbB4 activation at the biochemical level in cultures by immunocytochemistry for pErbB4. We also used an anti-ErbB4 antibody, rather than anti-PSD95, for ErbB4-PSD95 co-immunoprecipitation in order to exclude any non-interneuronal confounding factors (for example, possible changes in PSD95 expression in pyramidal neurons). It is also difficult to definitively establish cell-autonomy *in vivo*. Although the titre of the virus used did not produce universal infection of PV interneurons, it is possible that infected interneurons were close enough to influence each other. However, we believe that this scenario is unlikely because pyramidal neurons are considered the major source of NRG1 expression and signalling in the cortex^44^.

Recent studies suggest that loss of function of DISC1 in developing interneurons leads to impaired inhibition in the mature cortex, an opposite effect from our findings of enhanced interneuronal excitation and inhibitory synapse formation. However, it is important to note that these studies either use genetic mutant models of DISC1 (ref. 45), which do not specifically address the function of interneuronal DISC1, or knock down of DISC1 expression during neurodevelopment^26^, which may confound studies of the role of DISC1 in the adult brain. In the present study, our goal was to modulate DISC1 in a subset of cells at a particular time point, independent from the broader context of DISC1 functioning *in vivo*. To do this, we use Cre-dependent viral shRNA expression to specifically target interneurons in adulthood. This approach allows normal DISC1 expression during development and only knocks down DISC1 in mature interneurons. We are thus able to study a previously uncharacterized pool of DISC1 in cortical interneurons of the mature brain *in vivo*. Because the time point at which interneuronal function is disrupted has been shown to affect cognitive outcomes^46^, we believe that an approach that can distinguish adult brain function from early neurodevelopmental influences is important. The value of studying mechanisms specific to developmental stages can be appreciated by a recent study showing that disruption of ErbB4 mainly affected the final stages of glutamatergic synapse formation onto interneurons^8^.

Several studies on post-mortem patient brains have shown an increase in NRG1 and ErbB4 protein and mRNA levels^47^,^48^,^49^. In addition, transgenic mice overexpressing NRG1 (analogous to an ErbB4 gain of function) show schizophrenia-like phenotypes^50^,^51^,^52^. Our results are consistent with these lines of evidence, which indicate that upregulation of NRG1-ErbB4 signalling underlies mechanisms relevant to schizophrenia. However, we need to note that loss of function of NRG1-ErbB4 signalling in rodent models also display schizophrenia-like phenotypes^53^,^54^. Thus, we propose that ErbB4 signalling needs to be in an optimal range for proper network functioning and deviations above or below this range result in behavioural outcomes relevant to schizophrenia. This idea is reminiscent of the fact that both upregulation and downregulation of dopamine signalling result in disturbance of cognitive functions^55^,^56^.

A recent study of NRG1-ErbB4 signalling in interneurons has indicated another potential site of cross-talk with DISC1. ErbB4 reportedly interacts with and phosphorylates Kalirin-7 (Kal7), enhancing Rac1 activation and neurite outgrowth in response to NRG1 (ref. 57). Given our current finding that DISC1 reduces NRG1-induced ErbB4 activation, we predict that DISC1 might also reduce Kal7-Rac1 signalling. This is broadly consistent with earlier findings from our group, which show that DISC1 impairs Kal7-Rac1 signalling by tethering Kal7 to PSD95 (ref. 20). Further studies that specifically target the excitatory postsynaptic density on mature interneurons will be necessary to fully understand the interactome responsible for the proper functioning of these cells.

Human studies have demonstrated interneuron deficits in schizophrenia by neuropathology and clinico-physiology (for example, oscillations)^58^,^59^. Independently, human studies also have indicated pyramidal neuron deficits by neuropathology and genetics^60^,^61^. Nonetheless, it is unclear what molecular mechanisms underlie the overall pathology affecting both interneuron and pyramidal neurons defects. Our present study focusing on interneurons, together with recent studies focusing on pyramidal neurons^20^, may provide the NRG1-ErbB4-DISC1 pathway as a basis for common driver to bridge pyramidal/interneuronal pathology in schizophrenia. Future studies that integrate these findings into the broader context of DISC1 functioning may provide insight into the convergence of genetic disruptions in mental illness.

## Methods

### Reagents

Antibodies used in this study were as follows: rabbit anti-phospho-ErbB4 (Cell Signaling Technologies, 1:1,000, western blotting), rabbit anti-phospho-ErbB4 (Abcam, 1:2,000, cell staining), rabbit anti-ErbB4 (Santa Cruz Biotechnology, 1:400), rabbit anti-NRG1 (Santa Cruz Biotechnology, 1:400), rabbit anti-DISC1 (mExon3 (ref. 62), 1:400), mouse anti-PV (Millipore, 1:500), mouse anti-GAD67 (Millipore, 1:500), goat anti-GFP (Sigma, 1:500), mouse anti-PSD95 (Millipore, 1:2,000), rabbit anti-phospho-tyrosine (Santa Cruz Biotechnology, 1:400), rabbit anti-phospho-Erk (Cell Signaling Technology, 1:1,000), rabbit anti-HA (BD Biosciences, 1:1,000), rat anti-RFP (Chromotek, 1:500), rabbit anti-GAD65 (Millipore, 1:500).

### Recombinant protein production and treatment

Recombinant NRG1-β-GST was produced as previously described^32^. HEK-293 cells (American Type Culture Collection) were treated with 10 nM NRG1 for 5 min at 37 °C. Primary cortical neurons were treated with 5 nM NRG1 for 15 min at 37 °C. Following treatment, cells were placed on ice, washed with ice-cold PBS, scraped into lysis buffer and sonicated.

### Recombinant DNA production

The shRNA sequence used to knock down endogenous DISC1 was: 5′-GGCAAACACTGTGAAGTGC-3′ (refs 21, 33, 34, 35). This hairpin fragment was subcloned into a lentiviral vector previously used by our group^32^. Oligonucleotides containing the shRNA sequence were synthesized, annealed and digested using *Xho*I and *Eco*RI for insertion into a published AAV construct containing a floxed-stop codon cassette to allow Cre-dependent shRNA expression^63^. Following ligation, One Shot Stbl3 Chemically Competent *E. Coli* (Invitrogen) were transformed and spread on Luria Bertani agar plates containing ampicillin to select positive colonies. Plasmid DNA for all viral constructs was prepared using Endofree Plasmid Maxi kits (Qiagen). All plasmids for transfection into HEK-293 cells were prepared using Plasmid Maxi kits (Qiagen).

### Virus preparation

Lentivirus was produced by co-transfection of established lentiviral plasmids^20^ and packaging vectors (RRE, Rev, VSVG) into HEK-293FT cells (Invitrogen). Transfection was performed using Lipofectamine 2000 reagent (Invitrogen) following the manufacturer's protocol. 48–72 h after transfection, supernatants were collected and virus was concentrated following our previously described protocol^32^. AAV was also generated as previously described^63^. Briefly, AAV plasmid and helper constructs were co-transfected into HEK-293 cells using CaCl_2_; supernatant and cells were collected 48–72 h later. Virus was precipitated using (NH_4_)_2_SO_4_, then the precipitate was re-suspended and purified on an iodixanol gradient by ultracentrifugation. Further concentration of virus was done using an Amicon Ultra-100 K filter unit (Millipore).

### Cell culture and transfection

HEK-293 cells were cultured as previously described^32^. Transfection of HEK-293 cells for non-virus experiments was done using Polyfect reagent (Qiagen) following the manufacturer's protocol. Dissociated primary cortical neurons from rats (E19, Sprague–Dawley, Charles River) were also cultured as previously described^20^,^32^. Plasmid DNA for transfection was produced using the EndoFree Maxiprep kit (Qiagen), and transfection was performed using Lipofectamine 2000 reagent (Invitrogen) following the manufacturer's protocol.

### Generation of Disc1 LI model

The mouse locus containing *Disc1* is closely syntenic to the human locus. It includes the *Tsnax*/*Trax* gene located at 5′ to the *Disc1* gene. The 40-kb locus that we deleted covers exons 1, 1b, 2 and 3, as well as depletes *Tsnax*/*Trax-Disc1* intergenic splicing. We also deleted an miRNA in intron 1, which could alter functions and levels of DISC1, by targeting at least some DISC1-binding partner genes (such as 14-3-3, CRMP1/2). The targeting scheme that was applied to delete the *Disc1* locus was essentially as described^64^. Briefly, a bacterial artificial chromosome-based genomic library generated from C57BL/6J-129SvEv-hybrid embryonic stem (ES) cell lines (Regeneron) was screened to identify a *Disc1*-containing clone that we subsequently modified using a RecA-mediated recombination strategy to replace the genomic region (∼40 kb) with the neomycin selection cassette. The successfully modified bacterial artificial chromosome was electroporated into C57BL/6J-129SvEv hybrid ES cell lines and the targeted clones were identified by Velocigene screening followed by confirmation via conventional Southern blots. Among three successfully targeted ES clones injected into C57BL/6J blastocysts, two rendered highly chimeric males, which transmitted the mutation through the germline when crossed with C57BL/6J mice. F1 heterozygous offspring from both lines were sufficiently backcrossed with C57BL/6J to standardize the genetic background, and both mouse lines showed identical phenotypes. Although body weight was lower, no lethal phenotypes were observed in the mice.

### Stereotaxic injection

Stereotaxic injection into the prefrontal cortex (PFC) of adult mice was conducted similarly to a previously described protocol^20^. Adult mice (>P60, female) were anaesthetized with ketamine and immobilized in a stereotaxic frame. A scalp incision and small craniotomy were performed under sterile conditions. Virus was injected using a microinfusion pump and sharp glass pipette. Coordinates for injection into the PFC are: anterior-posterior (AP)+1.6 mm, medial lateral (ML)±0.5 mm, dorsal-ventral (DV)−1.8 mm. Following surgery, the incision was closed with sutures and the mouse was allowed to awaken completely before being returned to its cage. All experiments involving animals were performed in compliance with the Institutional Animal Care and Use Committee.

### Co-immunoprecipitation and western blotting

Co-immunoprecipitation was performed as previously described^32^,^65^. Protein lysates were prepared by cell scraping or tissue homogenization in lysis buffer (20 mM Tris (pH 7.4), 150 mM NaCl, 1 mM EDTA, 1 mM EGTA, 1% Triton X-100, 2.5 mM sodium pyrophosphate, 1 mM β-glycerolphosphate, 1 mM Na_3_VO_4_ and 1 mM Pefabloc (Roche Applied Science)). Lysates were pre-cleared with 25 μl Protein A/G Agarose beads (Chemicon) and 1 μg IgG, before incubation with precipitating antibody overnight at 4 °C. Samples were then incubated for 1 h with 50 μl Protein A/G Agarose beads, spun down and washed three or four times with lysis buffer or PBS, then boiled in sample buffer for 2–10 min. For western blotting, 20 μl of sample containing up to 50 μg total protein was loaded in a Novex 8% Tris-Glycine gel (Life Technologies) and run at room temperature, followed by transfer to a PVDF membrane (Thermo Scientific). Membranes were blocked for 1 h in 5% milk in PBS, incubated with primary antibody overnight, then with secondary antibody (goat anti-mouse or anti-rabbit HRP, Thermo Scientific) for 2 h at room temperature before exposure and quantification (see Supplementary Methods for details). For phospho-specific antibodies, tris-buffered saline (TBS) and 5% bovine serum albumin were used instead of PBS and 5% milk, respectively.

### Immunohistochemistry and immunocytochemistry

Mouse brains were perfused with 4% paraformaldehyde, cryoprotected using 20 and 30% sucrose in PBS, mounted in optimal cutting temperature reagent and sectioned at 20 μm thickness using a cryostat. Frozen sections or cultured cells on coverslips were fixed with 3.7% formaldehyde for 10 min at room temperature. Sections or coverslips were blocked with TBS containing 1% BSA, 5% normal goat serum and 0.2% Triton X-100 followed by incubation in primary antibody overnight at 4 °C, washing with TBS, incubation in secondary antibody for 2 h at room temperature, TBS washing and labelling with 4,6-diamidino-2-phenylindole (Amersham, 1:10,000) and mounting using ProLong Gold antifade reagent.

### Electrophysiology

Electrophysiological recordings from acute cortical slices were performed as previously described^66^. Briefly, fluorescent illumination was used to target GFP-positive cells in layer 3 of PFC and whole-cell recordings were obtained with Multiclamp 700B amplifiers (Molecular Devices). Cortical slices of 300 μm thick were perfused at room temperature (27 °C) in artificial cerebrospinal fluid containing 119 mM NaCl, 2.5 mM KCl, 2 mM CaCl_2_, 2 mM MgCl_2_, 26 mM NaHCO_3_, 1 mM NaH_2_PO_4_ and 11 mM glucose and were gassed with 5% CO_2_/95% O_2_ (pH 7.4). Patch recording pipettes (3–5 MΩ) were filled with internal solution containing 115 mM cesium methanesulfonate, 20 mM CsCl, 10 mM HEPES, 2.5 mM MgCl_2_, 4 mM Na_2_ATP, 0.4 mM Na_3_GTP, 10 mM Na-phosphocreatine and 0.6 mM EGTA (pH 7.25). mEPSCs were recorded in artificial cerebrospinal fluid including 1 μM tetrodotoxin and 100 μM picrotoxin (Sigma-Aldrich) and analysed using Mini Analysis Program (Synaptosoft, Inc.). Stable recordings were obtained for at least 10 min and 350 events per cell were used for analysis. At least 20 cells from four or more animals were analysed per condition.

### Subcellular fractionation

Fractionation of mouse brains was performed as previously described^67^. Briefly, dissected cortices were dounce homogenized in TEVP buffer (10 mM Tris-HCl, pH 7.4, 5 mM NaF, 1 mM Na_3_VO_4_ and complete protease inhibitor (Roche)) with 320 mM sucrose and centrifuged at 1,000*g* for 10 min to obtain pellet P1 and supernatant S1. S1 was centrifuged at 10,000*g* for 10 min to obtain P2 and S2. P2 was lysed hypo-osmotically and centrifuged at 25,000*g* for 1 h to obtain pellet LP1. S2 was centrifuged at 165,000*g* for 1 h to obtain P3 and S3. LP1 and P3 were re-suspended in TEVP and sonicated once.

### GAD65 bouton measurement

GAD65 bouton formation was measured similarly to published protocols^68^. Briefly, perfused cortical sections were stained with GFP, RFP and GAD65, and confocal z-stacks containing labelled cells were captured at × 63 magnification. Stacks were imported into ImageJ and images of pyramidal neurons in which the optical plane contains the largest soma profile were quantified. Pyramidal neurons (visually defined by RFP) were traced and an area within 2 μm of the cell surface is analysed for GAD65 immunoreactive punctae, with area between 0.15 and 2.5 μm^2^ (ref. 69). GAD65 signal, not GFP, was used to define boutons. Quantification was done blind to the shRNA condition.

### Statistical analysis

The significance of inter-group differences was calculated using GraphPad Prism 5 software. For analyses of two groups, *P*-values are determined by *t*-test with equal variances or *t*-test with unequal variances after variances of two groups were tested by *F*-test. For analyses of three or more groups, *P*-values were determined by one-way analysis of variance followed by Bonferroni *post hoc* corrections.

## Additional information

**How to cite this article:** Seshadri, S. *et al.* Interneuronal DISC1 regulates NRG1-ErbB4 signalling and excitatory–inhibitory synapse formation in the mature cortex. *Nat. Commun.* 6:10118 doi: 10.1038/ncomms10118 (2015).

## Supplementary Material

Supplementary Information Supplementary Figures 1-12, Supplementary Methods and Supplementary References.
