# Supplementary material for: Interneuronal DISC1 regulates NRG1-ErbB4 signalling and excitatory–inhibitory synapse formation in the mature cortex
Source: Nat Commun. 2015 Dec 11;6:10118. doi: 10.1038/ncomms10118 (PMC4682104; doi:10.1038/ncomms10118)
Supplement: Supplementary Information — Supplementary Figures 1-12, Supplementary Methods and Supplementary References. [file ncomms10118-s1.pdf]

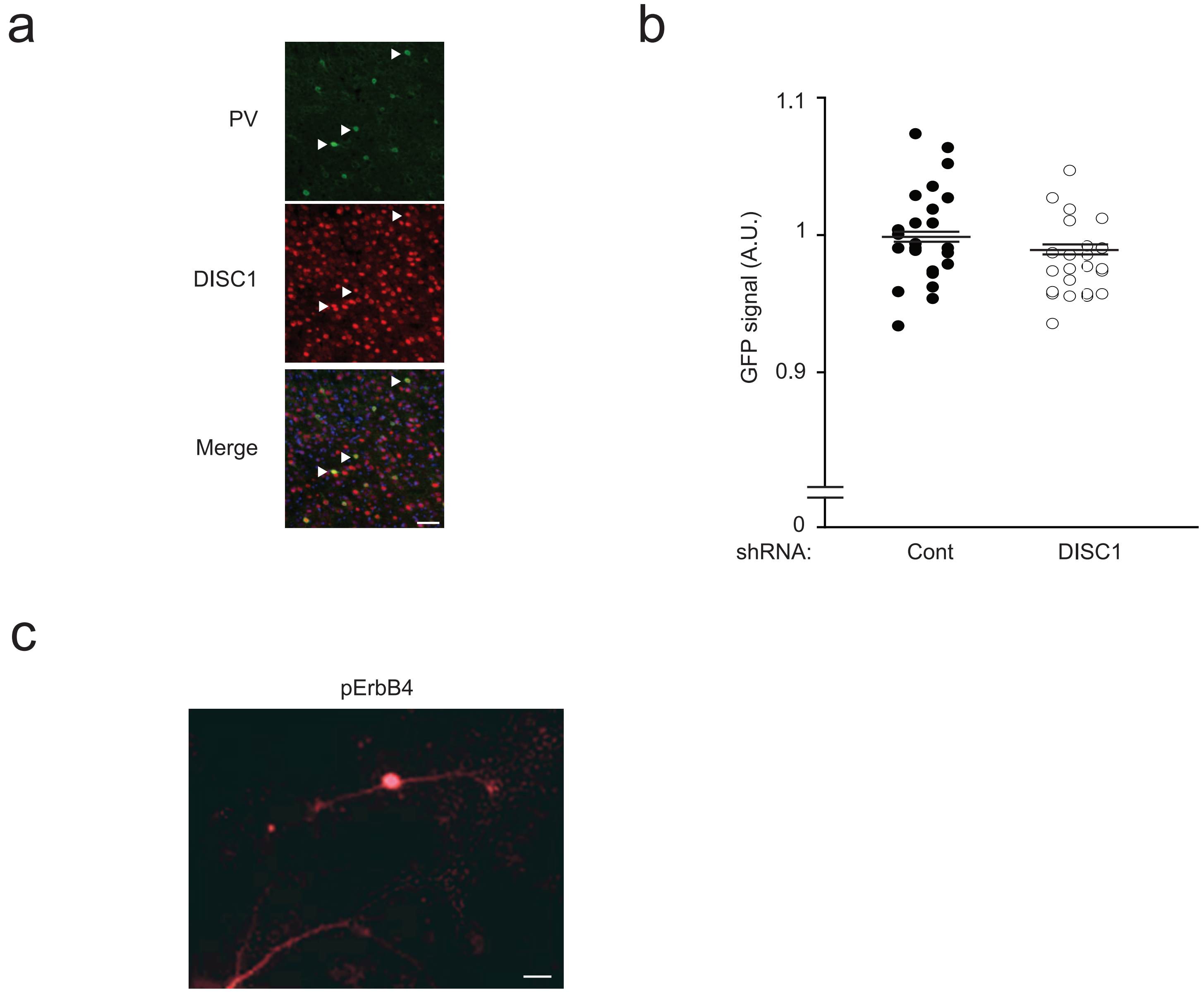

Supplementary Figure 1: Gene expression in PV interneurons (a) Immunohistochemistry for PV and DISC1 (anti-DISC1 antibody mExon3) in cortical sections from adult WT mice. DISC1 is expressed in PV-positive cells (arrowheads). Scale bar, 20 μm. (b) GFP levels were not significantly different in cells transfected with Cont or DISC1 shRNA. (c) pErbB4 expression pattern by immunocytochemistry. pErbB4 expression was observed in the cell body as well as processes (brightness-enhanced left panel from Fig. 1b). Scale bars, 10 μm. N = 22 cells (Cont shRNA) or 24 cells(DISC1 shRNA) from 3 independent experiments.

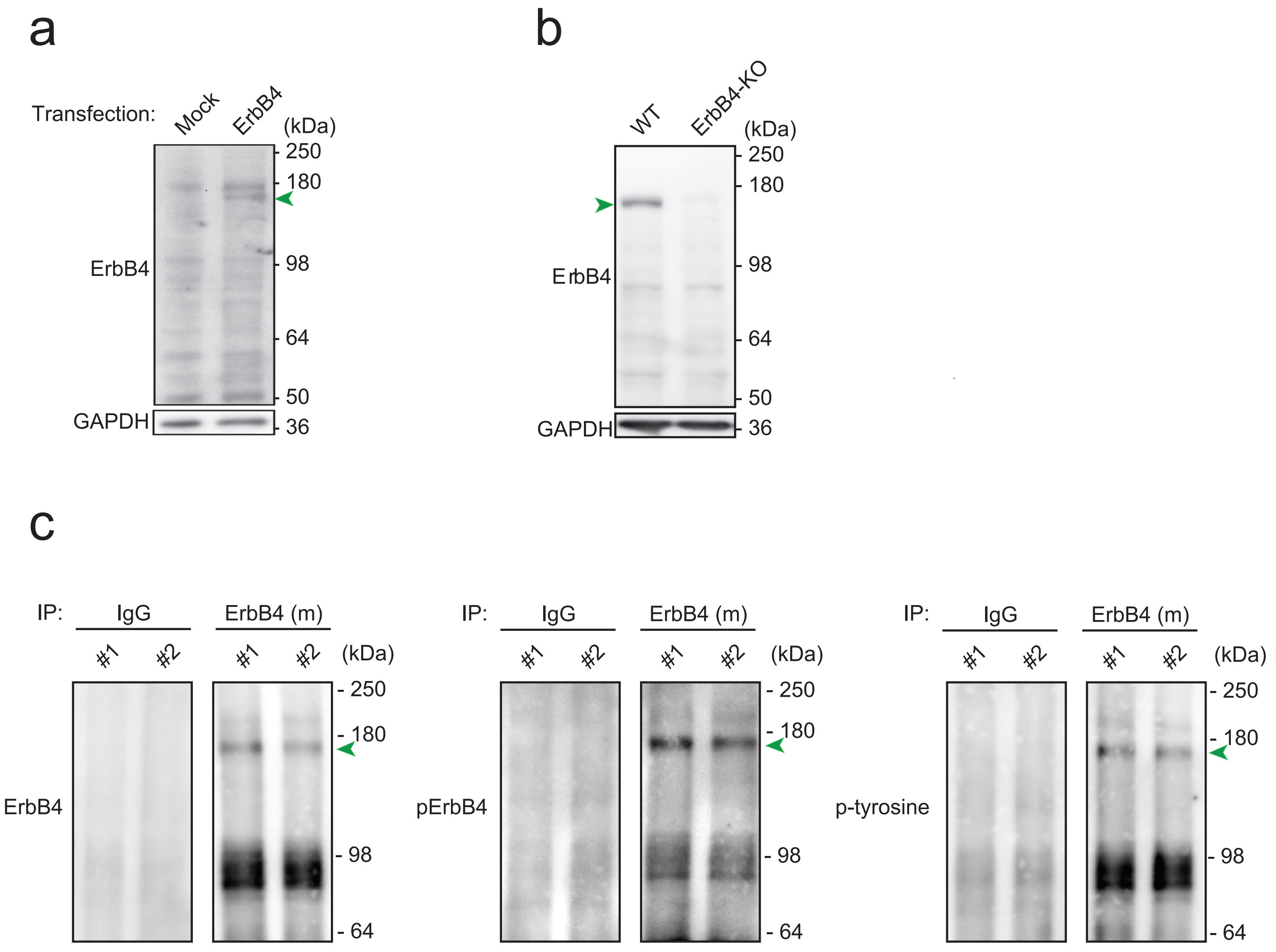

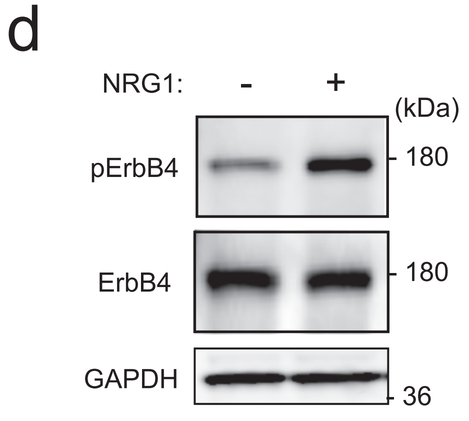

Supplementary Figure 2: Validation of key antibodies (a) Western blot for ErbB4 in HEK293 cells transfected with ErbB4 or mock transfected. The absence of a band in mock transfected cells confirmed specificity of the antibody (arrow). (b) Western blot for ErbB4 in cortical lysates from ErbB4-KO or WT mice. The absence of a band in ErbB4-KO brains confirmed specificity of the antibody (arrow). (c) Western blot for ErbB4 (left), pErbB4 (middle), and phospho-tyrosine (right) in protein lysates from NRG1-treated mature primary cortical neuron cultures precipitated with monoclonal ErbB4 or mouse IgG. Two trials were performed for each experiment. Detection of a band corresponding to ErbB4 at 180kDa was confirmed for all three antibodies (arrow). (d) Western blot for pErbB4 in lysates from primary cortical neuron cultures treated with a recombinant peptide containing the EGF-like domain of NRG1. Activation of ErbB4 was increased following NRG1 treatment in vitro. Total levels of ErbB4 were unaffected. GAPDH, loading control.

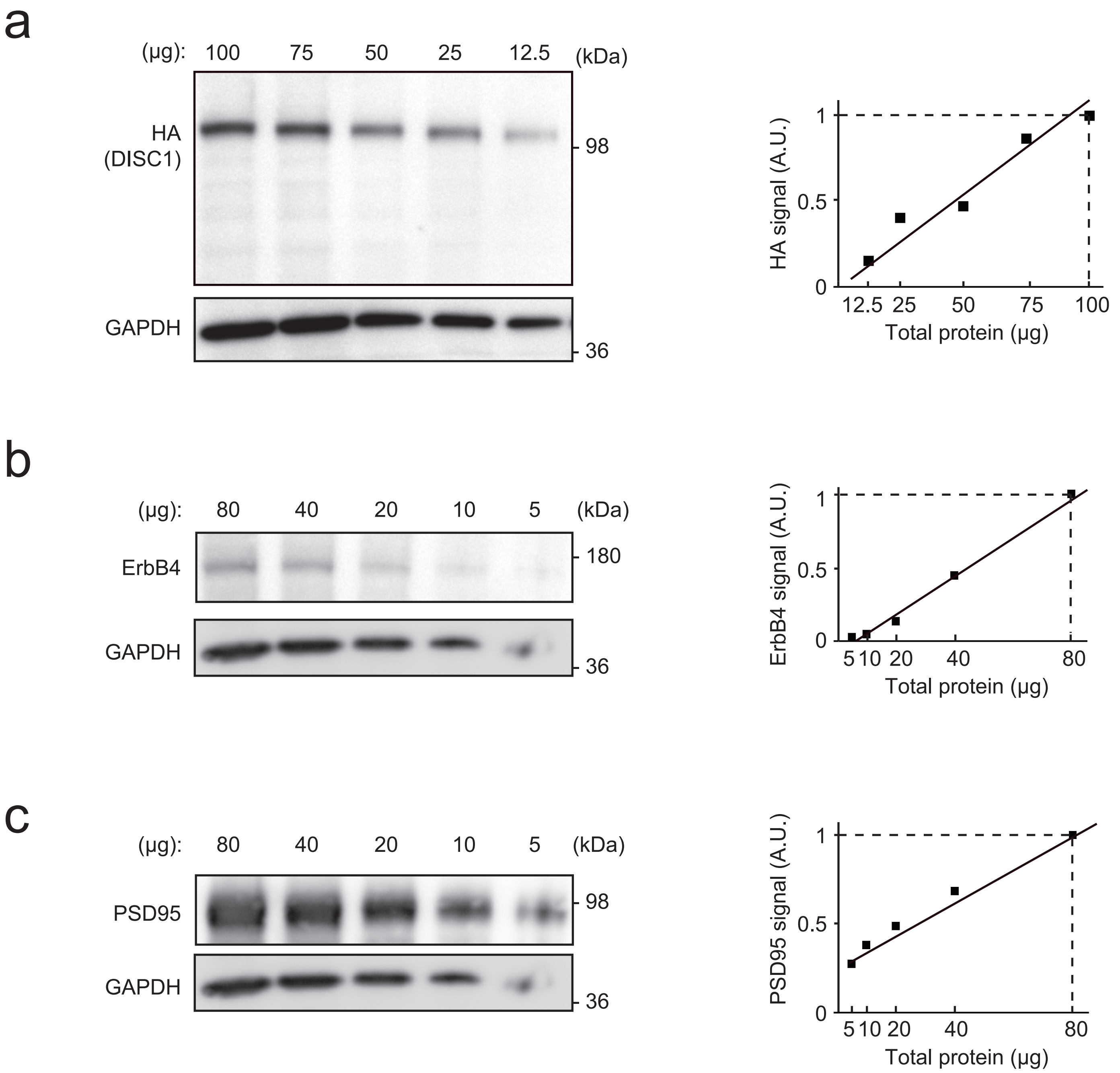

Supplementary Figure 3: Validation of key antibodies (a) Western blot for HA in lysates from DISC1-HA transfected HEK293 cells. Signal intensity vs. protein loaded was plotted to obtain a standard curve. (b) Western blot for ErbB4 in cortical lysates from WT mice. Signal intensity vs. protein loaded was plotted to obtain a standard curve. (c) Western blot for PSD95 in cortical lysates from WT mice. Signal intensity vs. protein loaded was plotted to obtain a standard curve.

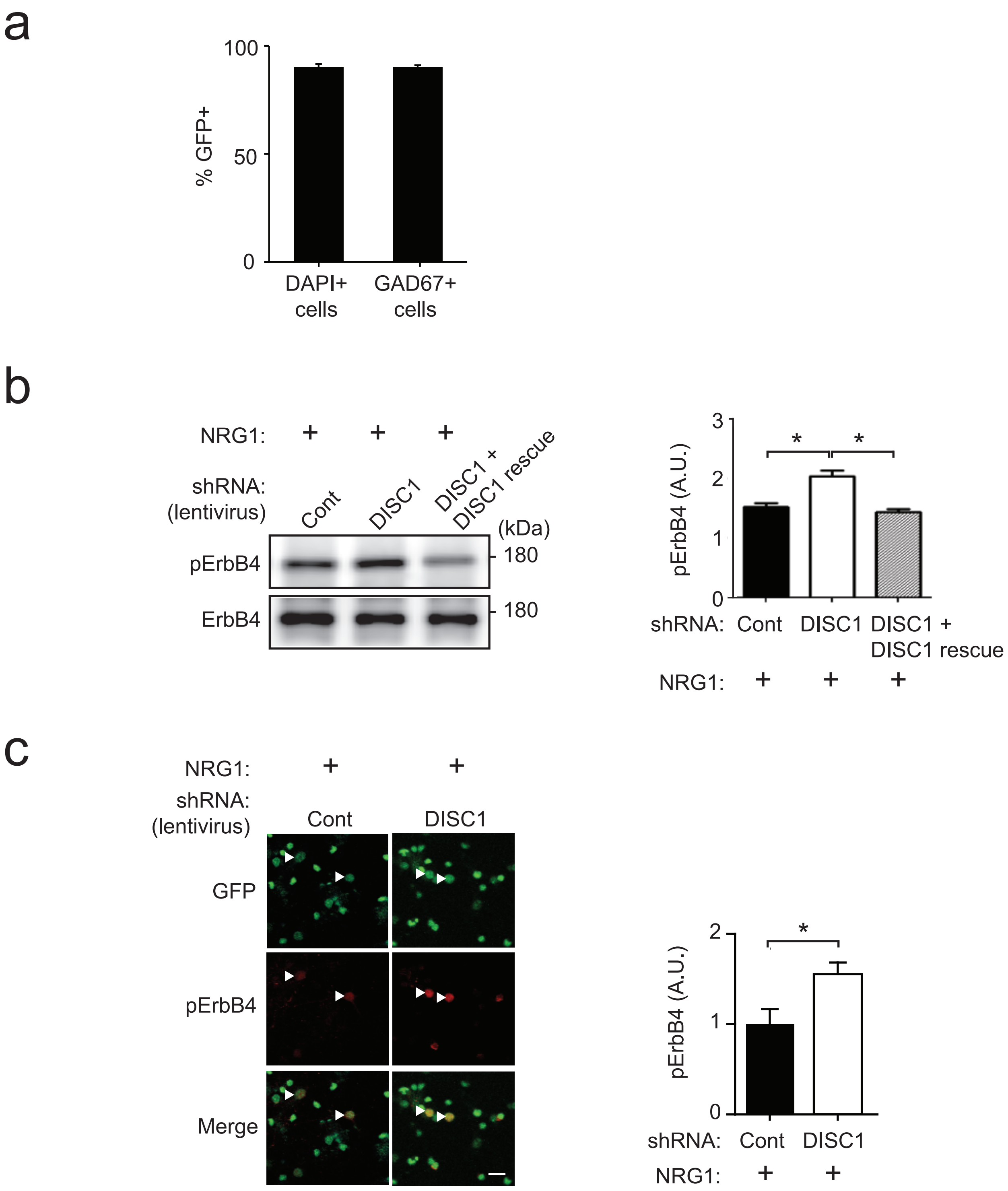

Supplementary Figure 4: DISC1 knockdown enhances ErbB4 activation in vitro (a) Infection efficiency of lentivirus in mature primary neuron cultures. GAD67-positive (GAD67+) cells (i.e. interneurons) were efficiently infected by lentivirus in vitro (N = 5 cultures). (b) Western blot for pErbB4 and ErbB4 in lysates from primary neuron cultures treated with NRG1 following infection with a lentivirus expressing shRNA (Cont or DISC1) or a lentivirus expressing DISC1 shRNA and shRNA-resistant DISC1 ('DISC1 rescue'). DISC1 knockdown enhanced NRG1-induced ErbB4 activation. Rescuing DISC1 expression abolished this effect. (c) Immunohistochemistry for pErbB4 in primary cortical neuron cultures infected with shRNA (Cont or DISC1) and treated with NRG1. Non-cell-type-specific DISC1 knockdown enhanced NRG1-induced ErbB4 activation. *, $p<0.05$; scale bar, 20 μm. Blue signal in images represents DAPI stain. All data are represented as mean±s.e.m. N = 30 cells per group from 3 independent experiments.

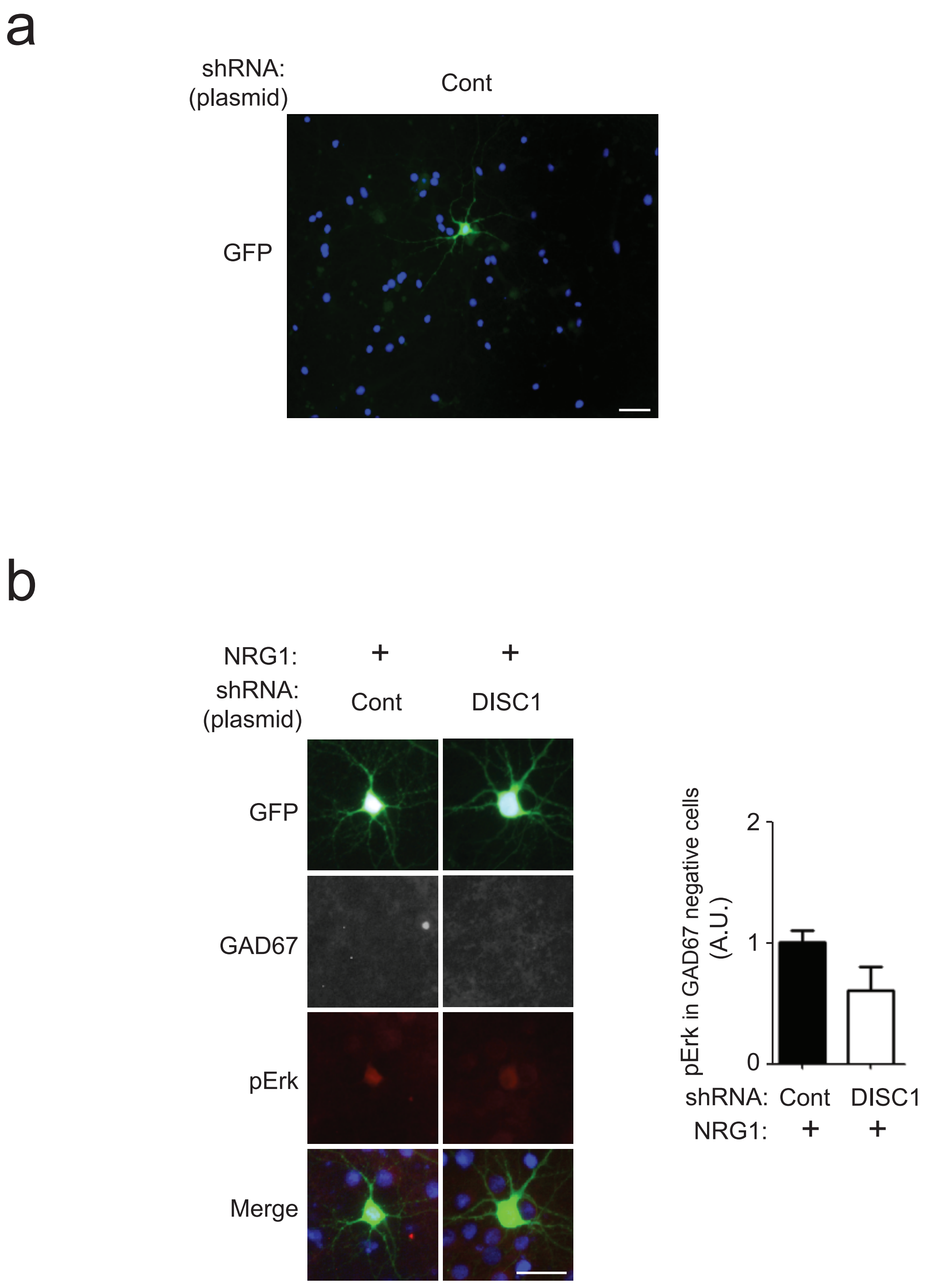

Supplementary Figure 5: Cell-autonomous targeting of DISC1 by low-efficiency transfection (a) GFP expression in mature primary cortical neurons transfected with shRNA (Cont, GFP co-expressed). A transfected cell is surrounded by unaffected cells. (b) Immunostaining for pErk and GAD67 in mature primary cortical neurons transfected with shRNA (Cont or DISC1, GFP co-expressed) and treated with NRG1. pErk immunoreactivity in GAD67-negative cells was not significantly changed. scale bar, 10 μm. Blue signal in images represents DAPI stain. All data are represented as mean±s.e.m.

a

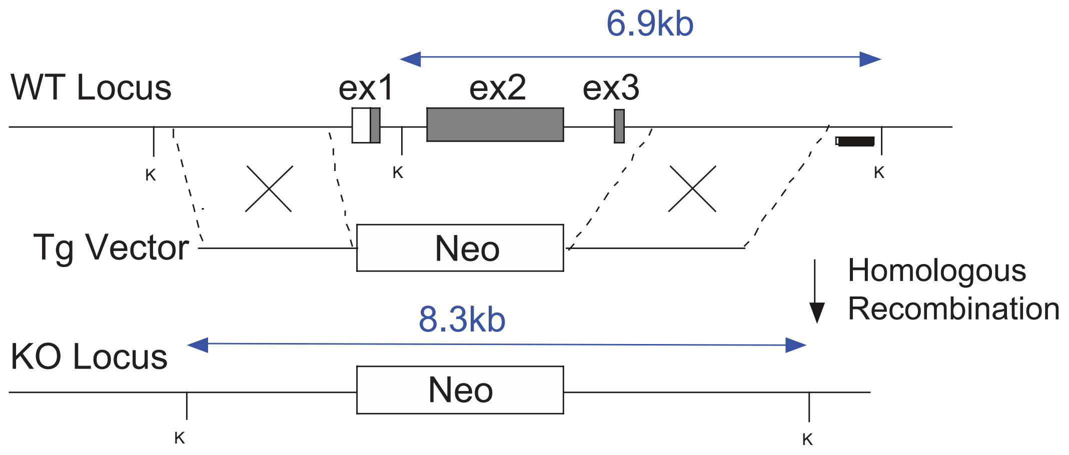

b

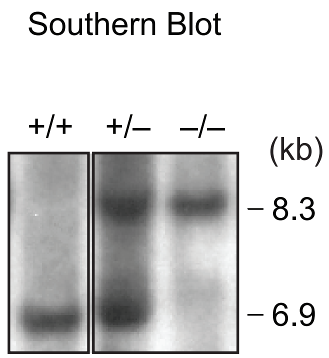

Supplementary Figure 6: Generation of Disc1-LI mice (a) Genetic targeting scheme for Disc1-LI mice. The first 3 exons of Disc1 (6.9 kb) were replaced by a Neomycin resistance cassette (8.3 kb) by homologous recombination. Mice were produced as described (Methods). (b) Loss of Disc1 exons was confirmed by Southern blotting.

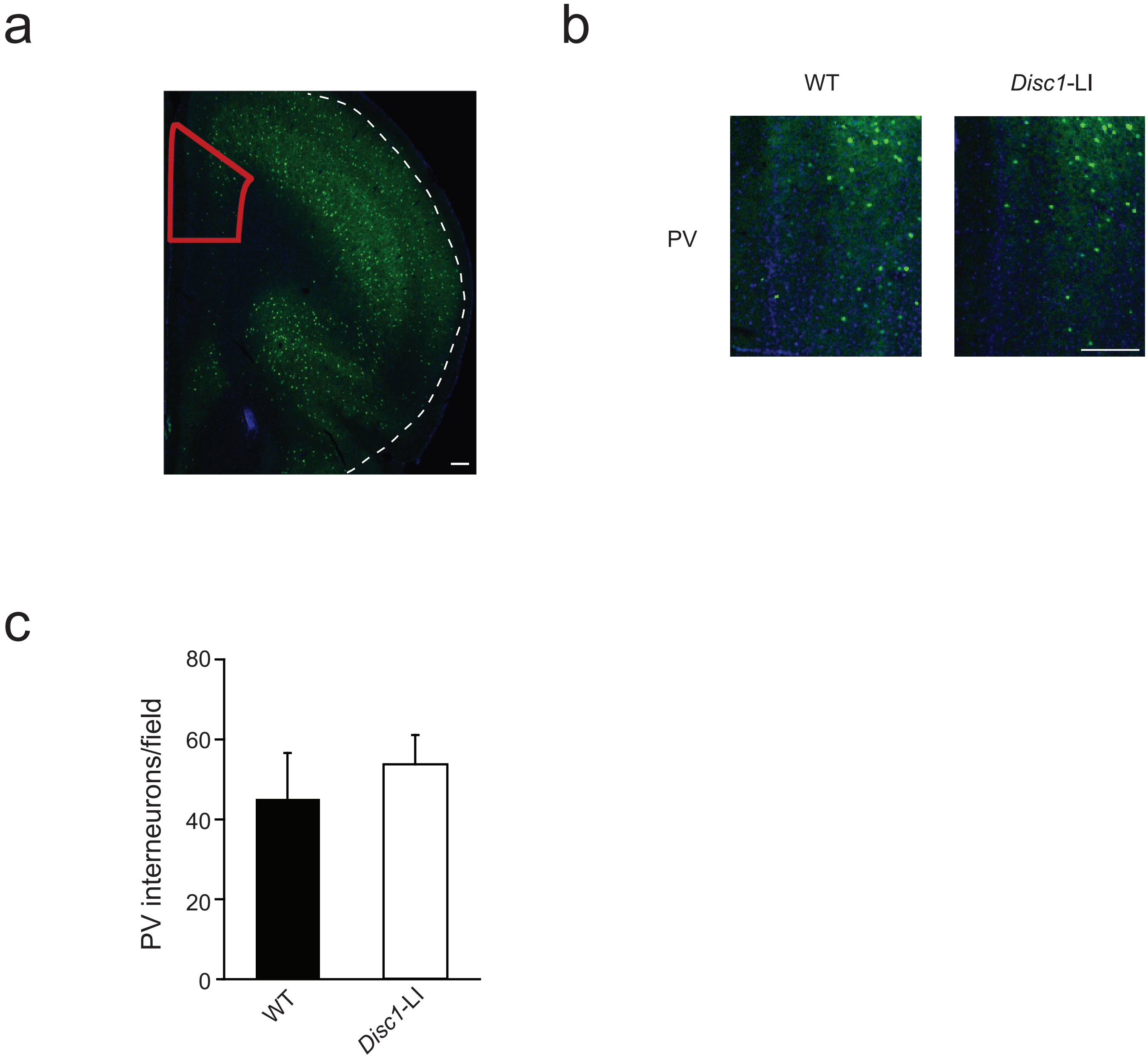

Supplementary Figure 7: PV interneurons in Disc1-LI mice (a) Immunohistochemistry for PV interneurons in mPFC. Region used for quantification is outlined (red box). Scale bar, 100 µm. (b) Representative cortical sections from adult Disc1-LI mice or WT littermates used for quantification of PV interneurons. Scale bar, 250 µm. (c) Total numbers of PV interneurons in mPFC were not significantly changed (N = 3 mice).

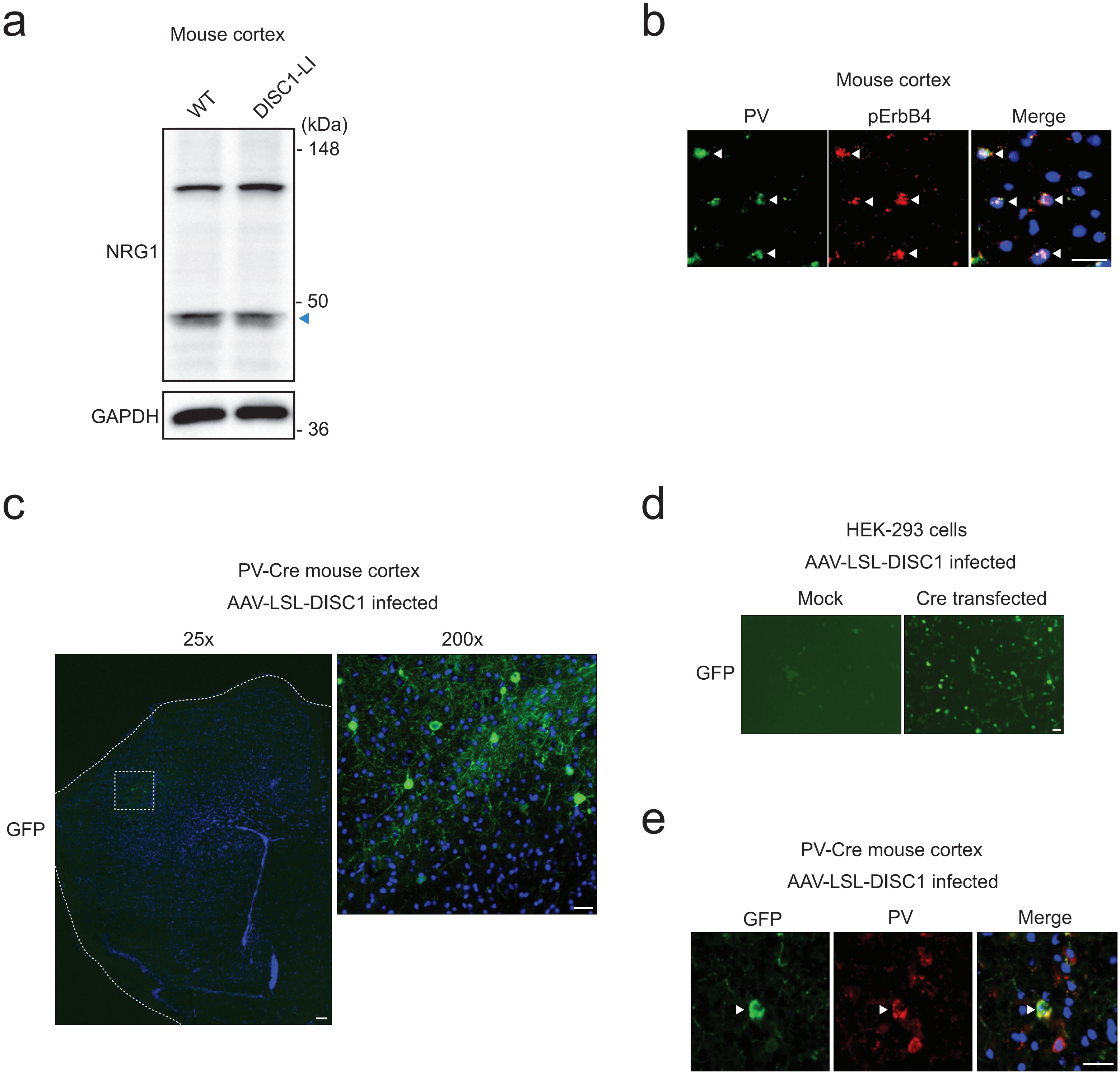

Supplementary Figure 8: Cre-dependent AAV mediated GFP expression (a) Western blot for NRG1 (arrowhead) in cortical lysates from adult DISC1-LI mice or WT littermates. No change in NRG1 expression was observed in DISC1-LI. (b) Immunohistochemistry for PV and pErbB4 in cortical sections from WT mice. pErbB4 was observed only in PV-positive cells (arrowheads). Scale bar, 20 μm. (c) GFP expression in cortical sections from PV-Cre mice injected with AAV-GFP. GFP expression was observed in the cortex 14 days post-injection. Scale bar, 200 μm (left panel), 20 μm (right panel). (d) GFP expression in HEK-293 cells transfected with Cre and infected with AAV-GFP. GFP expression was only observed in cells transfected with Cre, not in mock transfected cells. Scale bar, 20 μm. (e) Immunohistochemistry for GFP and PV in cortical sections from PV-Cre mice injected with AAV-GFP. GFP expression was found exclusively in PV-positive (i.e., Cre-expressing) cells. Scale bar, 20 μm. Blue signal in images represents DAPI stain.

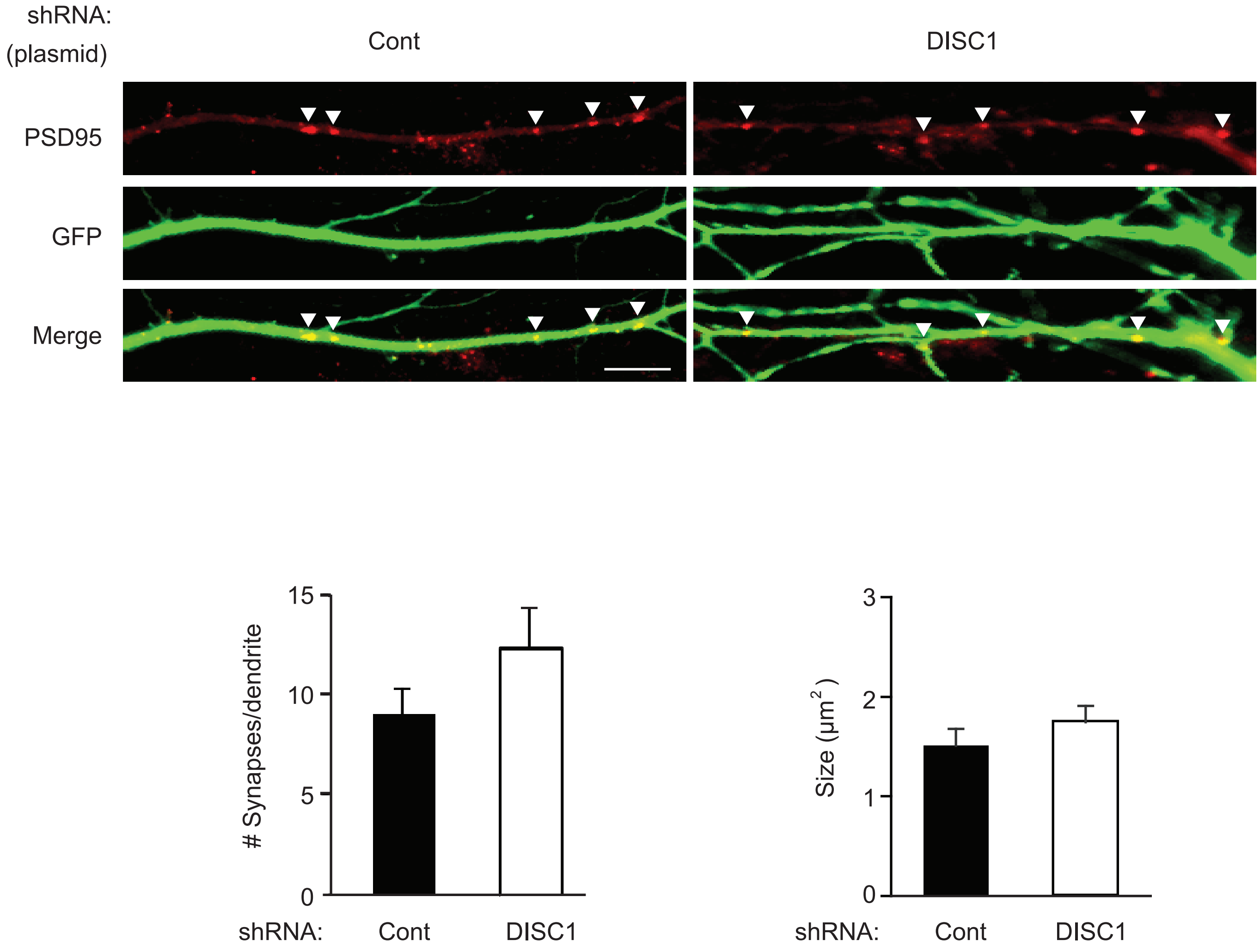

Supplementary Figure 9: Synaptic PSD95 in interneurons with DISC1 knockdown *in vitro*
Immunofluorescent cell staining for PSD95 (red) in mature primary cortical neurons transfected with shRNA (Cont or DISC1) co-expressing GFP (green). Cells were co-stained with GAD67 to select interneurons for analysis. Size and density of PSD95-positive punctae on GAD67-positive interneurons were quantified. No significant changes were observed in synaptic PSD95 expression following DISC1 knockdown. Scale bar, 5 μm. N = 15-16 dendrites from 3-4 cells per group.

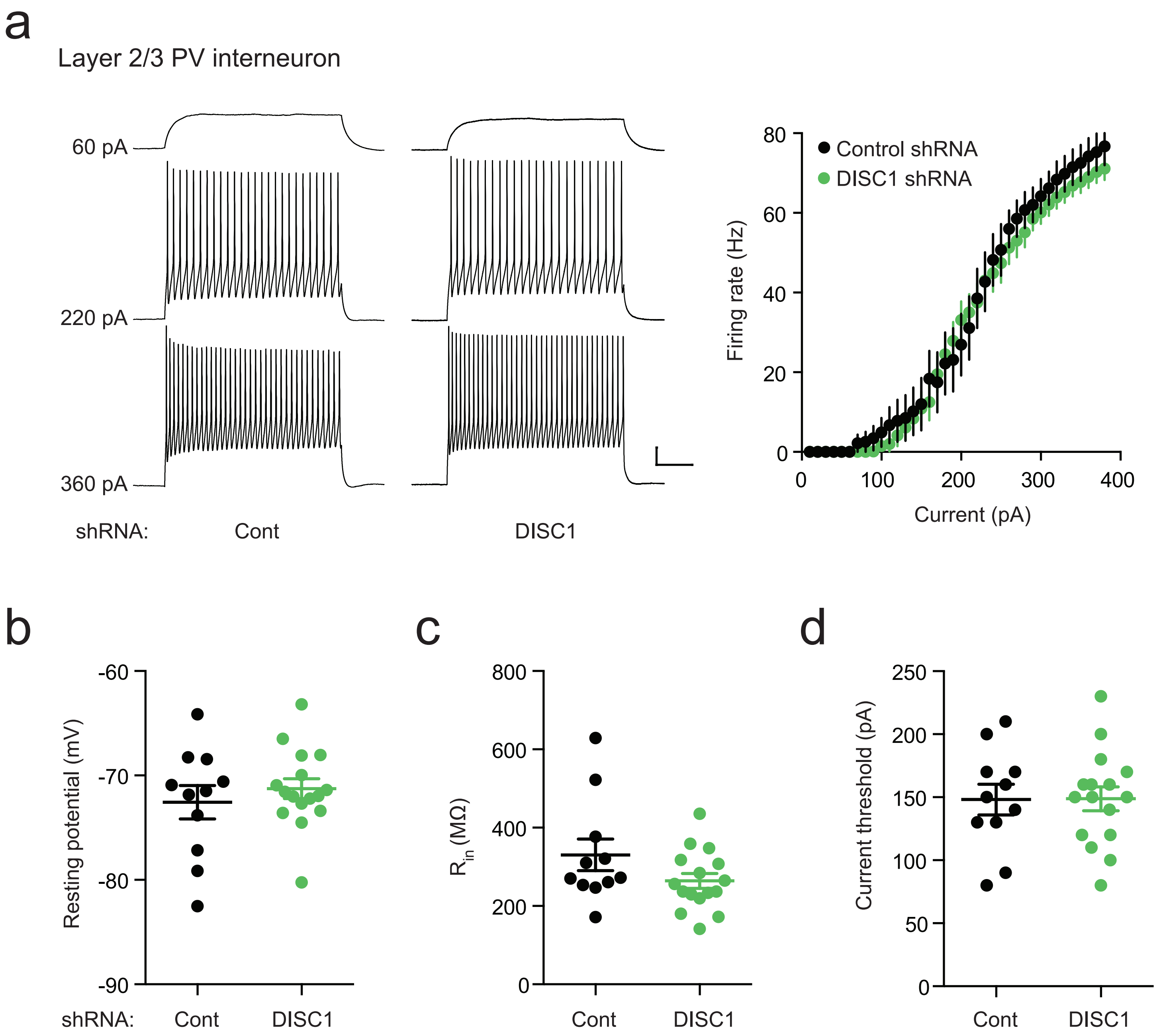

Supplementary Figure 10: Electrophysiological characterization of PV interneurons with DISC1 knock down. (a) PV-Cre mice were injected with Cre-dependent AAV expressing shRNA (Cont or DISC1). shRNA did not affect evoked firing rate in infected cells. (b) Resting potential was not changed by DISC1 knockdown. (c) Membrane input resistance was not affected by DISC1 knockdown. (d) Current threshold was not affected by DISC1 knockdown. All data are represented as mean±s.e.m. N = 11 cells (Cont shRNA), 16 cells (DISC1 shRNA).

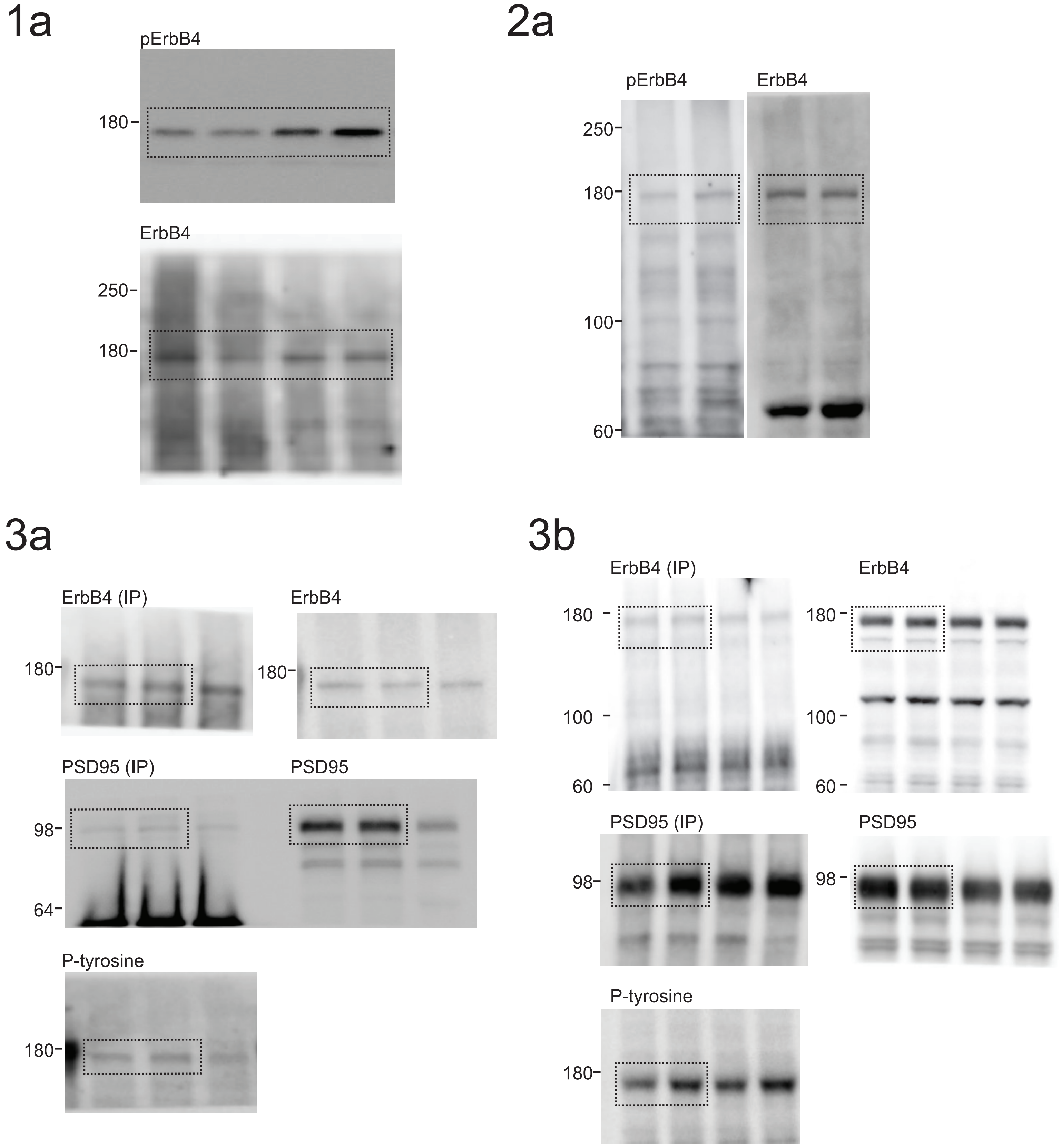

Supplementary Figure 11: Full versions of blots. Expanded and uncropped versions of representative Western blots shown in main figures. All numbers are ladder sizes in kDa. Panels are labeled with the corresponding main figure. Individual blots within a panel are labeled with the antibody for cross-reference with the figure.

3c

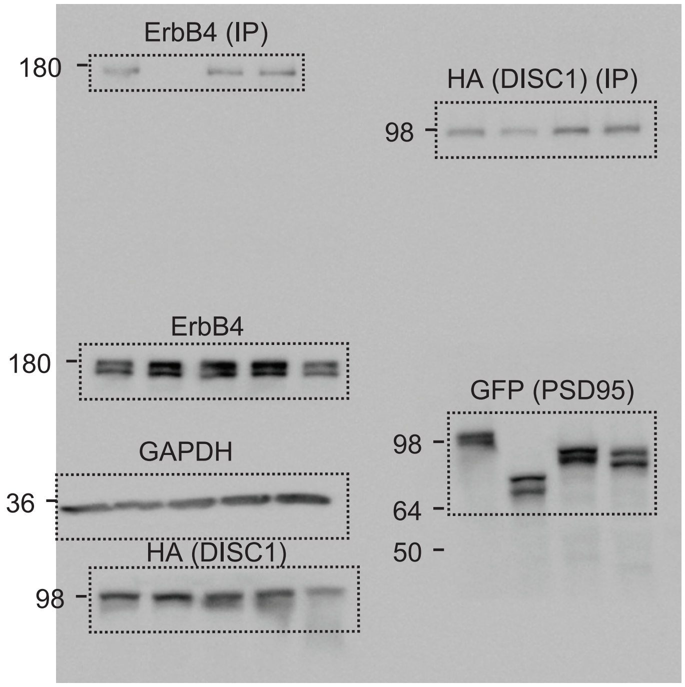

4a

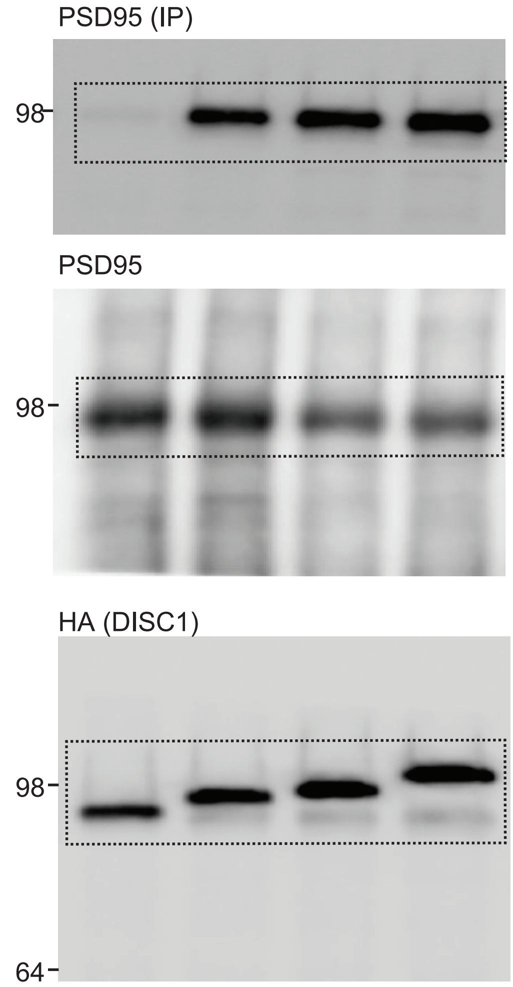

4c

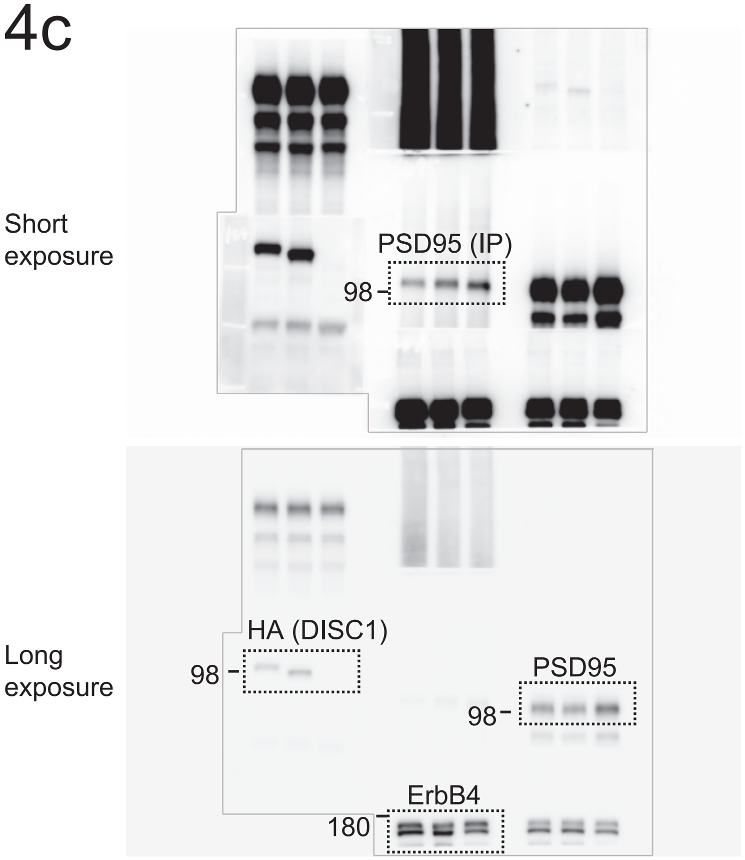

6a

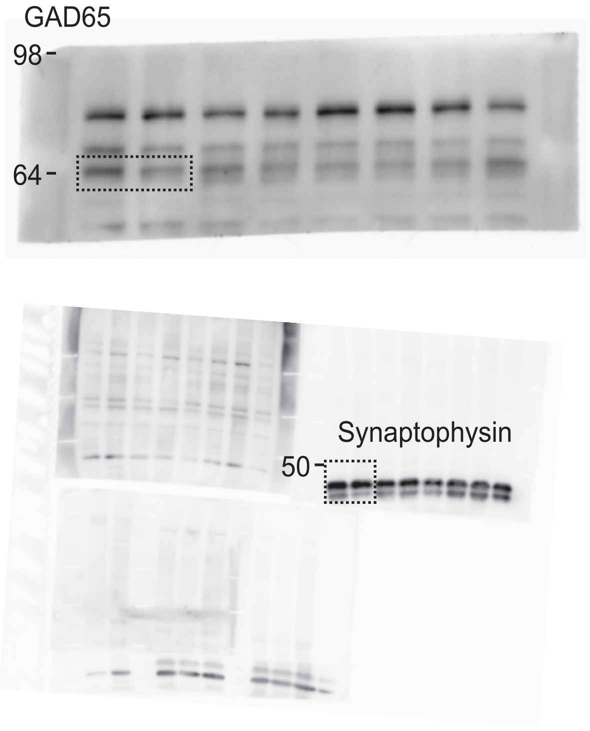

Supplementary Figure 12: Full versions of blots. Continued from Supplementary Figure 11.

Supplementary Methods

Number of samples per experiment: For all *in vitro* results, N reflects the combined count of quantified cells for at least 3 independent experiments (i.e. separate instances of cell culture, treatment, and immunofluorescent cell staining). Due to low transfection efficiency of our transfection reagent in primary neuron cultures (Lipofectamine 2000, Life Technologies), as well as low numbers of interneurons (~20% of cells), cell counts per condition per experiment were in the range of 5-15 cells; however the combined cell counts were at least 25 cells per condition. For all Western blotting results, experiments were repeated at least 3 times. For all immunohistochemistry results, brain sections from at least 3 individual mice were stained and quantified cells were pooled as above. All mice were older than 8 weeks at the time of sacrifice. All data was normalized before comparison across experiments. Numbers of animals and cells used for mEPSC recordings were as follows: DISC1 shRNA, 5 animals, 27 cells; Cont shRNA, 4 animals, 20 cells. Numbers of animals and cells used for intrinsic spike recordings were as follows: DISC1 shRNA, 2 animals, 16 cells; Cont shRNA, 3 animals, 11 cells.

Quantification of immunofluorescent signal intensity: Following immunofluorescent cell staining *in vitro*, images of labeled cells were obtained using a standard fluorescent microscope. Identical acquisition parameters (i.e. focus, excitation intensity, exposure time) were used for each fluorescent secondary antibody per experiment for all images. The relevant individual channel from the raw image was used for quantification (e.g. the red channel for Alexa 568). Signal intensity was quantified using Photoshop (Adobe) by drawing a region of interest (ROI) around the cell, as visualized by the cytosolic GFP. Intensity within this ROI was reported by the histogram tool with background signal measured by moving the ROI to an empty region of the image. This was subtracted prior to normalization to the control signal. For quantification of immunohistochemistry in brain sections, images of labeled cells were obtained with a confocal microscope (LSM 510, Zeiss) and a similar procedure was followed.

Quantification of Western blotting: Western blots were incubated with enhanced chemiluminescence substrate (ECL, Pierce) and exposures were done using an ImageQuant LAS 4000 Mini (GE) digital image acquisition system. Raw images were quantified using Photoshop (Adobe). Rectangular ROIs were drawn around selected bands and signal intensity was reported by the histogram tool. In each experiment, the same size ROI was used for all bands for every antibody. Background signal was measured by moving the ROI to an empty area of the blot, and subtracted from the

target signal prior to normalization to loading control signal. Blots were cropped, and in some cases, levels were adjusted for figure clarity. In these cases, image processing was always uniformly applied to all bands for each antibody.

DISC1 plasmid construction: DISC1 shRNA sequence used was as follows: 5'-GGCAAACACTGTGAAGTGC-3' [1,2]. A DISC1 construct resistant to this shRNA was generated by introducing three silent mutations in the target sequence by site-directed mutagenesis[3], and was subcloned into the lentiviral FUGW backbone for rescue experiments[4]. DISC1 constructs with truncations or deletions were produced previously by our group[2] or generated by PCR based on publicly available DISC1 sequence (GenBank: AF222980.1).

PSD95 plasmid construction: PSD95 deletion constructs were produced by inverse PCR as previously reported[5], based on original domain mapping of PSD95[6]. The deleted domains corresponded to the following amino acids (AA): PDZ1, AA65-151; PDZ2, AA160-246; PDZ3, AA313-393; SH3, AA435-495; GK, AA534-712.

Statistical analysis: To compare two groups, Student's two sample unpaired t-test was used. For three or more groups, one way ANOVA followed by Bonferroni post hoc was used. For electrophysiological data, the D'Agostino Omnibus test was used to test normality of the distribution. If not normally distributed, Mann-Whitney test was used, otherwise, two-tailed two sample t-test with Welch correction was used.

Supplementary References

1 Hayashi-Takagi, A. *et al.* Disrupted-in-Schizophrenia 1 (DISC1) regulates spines of the glutamate synapse via Rac1. *Nature neuroscience* **13**, 327-332 (2010).

2 Kamiya, A. *et al.* A schizophrenia-associated mutation of DISC1 perturbs cerebral cortex development. *Nature cell biology* **7**, 1167-1178, doi:10.1038/ncb1328 (2005).

3 Ho, S. N., Hunt, H. D., Horton, R. M., Pullen, J. K. & Pease, L. R. Site-directed mutagenesis by overlap extension using the polymerase chain reaction. *Gene* **77**, 51-59 (1989).

4 Shahani, N., Seshadri. S., Jaaro-Peled, H., Ishizuka, K., Hirota, Y., Wang, Q., Koga, M., Sedlak, T., Korth, K., Brandon, N., Kamiya, A., Subramaniam, S., Tomoda, T., Sawa, A. DISC1 regulates trafficking and processing of APP and Aβ generation. *Molecular psychiatry* (2014).

5 Hsueh, Y. P., Kim, E. & Sheng, M. Disulfide-linked head-to-head multimerization in the mechanism of ion channel clustering by PSD-95. *Neuron* **18**, 803-814 (1997).

6 Kim, E., Niethammer, M., Rothschild, A., Jan, Y. N. & Sheng, M. Clustering of Shaker-type K+ channels by interaction with a family of membrane-associated guanylate kinases. *Nature* **378**, 85-88, doi:10.1038/378085a0 (1995).
